# QSAR Models for Predicting Oral Bioavailability and Volume of Distribution and Their Application in Mapping the TK Space of Endocrine Disruptors

**DOI:** 10.3390/jox15050166

**Published:** 2025-10-15

**Authors:** Guillaume Ollitrault, Marco Marzo, Alessandra Roncaglioni, Emilio Benfenati, Olivier Taboureau, Enrico Mombelli

**Affiliations:** 1 Inserm U1133, CNRS UMR 8251, Université de Paris Cité, 75013 Paris, France; guillaume.ollitrault@inserm.fr; 2 Laboratory of Environmental Chemistry and Toxicology, Department of Environmental Health Sciences, Istituto di Ricerche Farmacologiche Mario Negri IRCCS, 20156 Milano, Italy; marco.marzo@marionegri.it (M.M.); alessandra.roncaglioni@marionegri.it (A.R.); emilio.benfenati@marionegri.it (E.B.); 3 Institut National de l’Environnement Industriel et des Risques (INERIS), 60550 Verneuil en Halatte, France; enrico.mombelli@ineris.fr

**Keywords:** QSAR, oral bioavailability, volume of distribution, endocrine-disrupting chemicals, Toxicokinetics

## Abstract

Toxicokinetic (TK) properties are essential in the framework of chemical risk assessment and drug discovery. Specifically, a TK profile provides information about the fate of chemicals in the human body. In this context, Quantitative Structure–Activity Relationship (QSAR) models are convenient computational tools for predicting TK properties. Here, we developed QSAR models to predict two TK properties: oral bioavailability and volume of distribution at steady state (VD_ss_). We collected and curated two large sets of 1712 and 1591 chemicals for oral bioavailability and VD_ss_, respectively, and compared regression and classification (binary and multiclass) models with the application of several machine learning algorithms. The best predictive performance of the models for regression (R) prediction was characterized by a Q^2^_F3_ of 0.34 with the R-CatBoost model for oral bioavailability and a geometric mean fold error (GMFE) of 2.35 with the R-RF model for VD_ss_. The models were then applied to a list of potential endocrine-disrupting chemicals (EDCs), highlighting chemicals with a high probability of posing a risk to human health due to their TK profiles. Based on the results obtained, insights into the structural determinants of TK properties for EDCs are further discussed.

## 1. Introduction

In the realm of drugs, pharmacokinetics (PK) refers to the characterization of the absorption, distribution, metabolism, and excretion of xenobiotics in an organism [1]. Toxicokinetics (TK) is closely related to pharmacokinetics (PK), as it involves the generation of PK data, either as part of nonclinical toxicity studies or through dedicated supportive studies to evaluate systemic exposure. Such analyses are largely used in pharmaceutical and chemical industries, as they are critical for gaining insights into the TK propensities of potential drug candidates and for assessing risks associated with environmental chemicals [2,3].

To limit the costs of such experiments and still provide relevant information for decision-makers in the field of drug discovery and chemical risk assessment, in silico models capable of predicting key TK/PK properties, notably oral bioavailability and volume of distribution (VD), are commonly developed for initial estimation [4].

Oral bioavailability characterizes the fraction of an orally administered drug that reaches the systemic circulation (F%). It is calculated based on the relationship between plasma chemical concentration and time after administration. Oral bioavailability is defined as the percentage of the dose area under the curve of the chemical concentration in the plasma after oral administration, divided by the dose area under the curve of the concentration of the drug in the plasma after intravenous administration [5]. This comparison yields information about the proportion of chemicals reaching the bloodstream since intravenous administration circumvents the digestive system and first-pass metabolism. High oral bioavailability can result in exposure to toxic compounds after intake, and low oral bioavailability for drugs can increase the required dose, with the associated risk of toxicity through accumulation and metabolites [6].

The volume of distribution (VD) measures the ability of a chemical to remain in plasma or to redistribute to other tissue compartments. VD is computed by considering the amount of a chemical in the body divided by the plasma concentration of the same chemical [7]. In the field of drug discovery, having a priori knowledge about VD assists in optimizing drug therapies, avoiding undesirable effects, and proposing effective treatments. Specifically, at a constant clearance rate, a chemical with a high VD will have a longer elimination half-life than one with a low VD [8], since the former will persist in tissues while being slowly released into the bloodstream. Therefore, knowing the VD of environmental chemicals is also important in the field of chemical risk assessment since these chemicals might remain longer in tissues, which could lead to accumulation in the human body and result in toxicity, especially for lipophilic drugs [6]. Different VD-related terms are commonly used, with the volume of distribution at steady state (VD_ss_) generally being the most relevant, as it is used to determine the VD associated with the steady-state dosing of the chemical. It is calculated during the phase called “steady state”, when the distribution and elimination phases are equal [7].

Several computational studies have been conducted to predict oral bioavailability [9,10,11,12,13,14,15,16] and VD_ss_, and many have used QSAR models [17,18,19,20,21]. Most existing models have focused on oral bioavailability using classification approaches, while regression models have been primarily developed for VD_ss_, notably using Lombardo et al.’s dataset [17]. In our work, we combined datasets from multiple sources, including a newly developed dataset from Liu et al. [22].

In this context, we decided to collect a large dataset of chemicals and to develop different modeling algorithms for regression, binary-class, and multiclass prediction for oral bioavailability and VD_ss_.

The most relevant models were then used to assess the TK properties of potential endocrine-disrupting chemicals (EDCs). The focus on this category of chemicals is motivated by the fact that EDCs can disrupt the endocrine system and cause cancer, metabolic disorders, neurocognitive functions, infertility, immune diseases, and allergies [23,24,25,26,27] by interfering with the estrogen, androgen, and thyroid hormone receptors, exerting steroidogenesis (ER, AR, and TR)-mediated effects [28]. Thus, predicting potential EDCs with high oral bioavailability and high VD_ss_ could be relevant for regulatory purposes.

To complement this work, we also applied an existing QSAR model to predict the elimination half-lives (t_1⁄2_) of EDCs. The elimination half-life is a key toxicokinetic parameter that reflects the time required for the concentration of a chemical in the body to decrease by half. This feature is crucial for assessing a compound’s persistence, bioaccumulation potential, and dosing frequency, making it an important factor in risk assessment and regulatory decision-making [29,30].

Finally, this large-scale analysis provides insights into the structural features that might be important in the determination of TK for EDCs, which is further discussed below.

## 2. Results

### 2.1. Data Distribution

Starting from the chemicals with experimentally known F% and VD_ss_, multiple datasets were designed. The number of chemicals in each dataset is reported in Table 1.

The work described herein relied on three datasets for training models to predict oral bioavailability. The first dataset contained 1213 chemicals and was used to train regression models. The second dataset was composed of 1307 chemicals and was used to train classification models with a 50% dichotomizing threshold. The third dataset consisted of 1244 chemicals and was used to train multiclass models. All models trained on the three datasets were then evaluated on a common set of 405 chemicals with known F% values.

For the VD_ss_ analysis, a single dataset containing 1167 chemicals was used to train regression and multiclass classification models. Two validation sets, one with 390 chemicals and the other with 34, were used. The first set was used to assess the overall performance of the trained model both with and without applying applicability domains, whereas the second set was used to compare the model’s predictive performance with that of published QSAR models for the same endpoints, given that it is a set of chemicals commonly used to compare the precision of QSAR models in the literature.

#### 2.1.1. Oral Bioavailability Data

**Figure 1:**
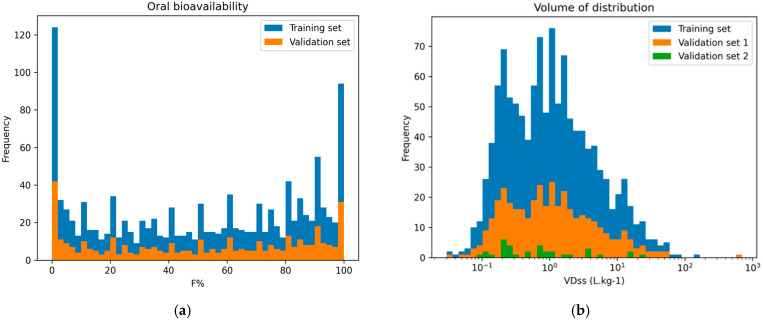
(**a**) Histograms of the distribution of oral bioavailability for the training set and the validation set for all chemicals with continuous F% values. (**b**) Distribution of the values characterizing VD_ss_ for the training set and validation sets 1 and 2.

The distributions of F% for the training and validation sets cover the complete endpoint range while having a similar shape, and therefore, they are suitable for model evaluation and training (Figure 1a). Indeed, the bioavailability values span the entire range from 0% to 100%. The distribution exhibits peaks at 0% and 100% bioavailability. This characteristic could be due to the limitations of oral bioavailability testing methods, as discussed by Aungst et al. [31]. The presence of many chemicals associated with 0% or 100%, with few in-between values, likely introduces a bias where the model yields correct predictions for the majority classes while displaying poor performance for intermediate values.

#### 2.1.2. Volume of Distribution Data

Figure 1b shows the distribution of VD_ss_ values across the training set, validation set 1, and validation set 2. To address the skewed nature of the VD_ss_ distribution (from 0.035 L·kg^−1^ to 700 L·kg^−1^) and facilitate model convergence, the dependent variable was logarithmically transformed, in base e, when regression models were applied. The distribution of VD_ss_ values across the training set, validation set 1, and validation set 2 is depicted in Figure 1b. We can observe that all three datasets exhibit comparable distributions across this range, ensuring coverage of VD_ss_ values for model training and evaluation.

#### 2.1.3. Chemical Space

The chemical space covered by the chemical sets was characterized using a Uniform Manifold Approximation and Projection for Dimension Reduction (UMAP) representation [32], facilitating a comprehensive exploration of the molecular landscape and enabling insightful analysis of the distribution of chemicals (Supplementary Figure S1).

UMAP on the oral bioavailability dataset (Supplementary Figure S1a) shows the distribution of chemicals while accounting for their F% values. The plot reveals that most points are distributed all around the two axes and exhibit a wide range of F% values, highlighting the difficulty of finding patterns between F% values and chemical similarity.

The UMAP representation of the ln(VD_ss_) values (Supplementary Figure S1b) projects the high-dimensional VD_ss_ dataset onto a two-dimensional map. The plot highlights the range of VD_ss_ values, with higher values on the left and lower values on the right, illustrating the relationship between chemical similarity and VD_ss_ values.

These observed patterns support the pertinence of using machine learning models for F% and VD_ss_ prediction. Machine learning algorithms can potentially learn effective predictive models that capture the diverse landscapes observed in these datasets.

### 2.2. Predictive Performance

#### 2.2.1. Oral Bioavailability Performance

Multiple models were trained and evaluated to predict oral bioavailability. Regression models were evaluated for the prediction of continuous values, while for binary-class and multiclass prediction, we imposed 50% and 30–60% thresholds. All models were evaluated using dedicated metrics.

From the 1826 molecular descriptors computed with Mordred, the most relevant ones were selected using the VSURF algorithm. This resulted in the selection of 66 molecular descriptors for a Topliss ratio (number of training chemicals per molecular descriptor) of 18:1 for the regression model, 59 molecular descriptors (Topliss ratio of 26:1) for binary classification prediction with a 50% threshold, and 70 molecular descriptors (Topliss ratio of 23:1) for multiclass prediction with 30–60% thresholds, with the Topliss ratio largely in compliance with the recommended threshold (>5) to avoid overfitting. Then, these selected molecular descriptors were utilized as input features to train the CatBoost, XGBoost, and RF models for predictive modeling.

The predictive performance of the algorithms was evaluated across regression (R), binary classification (BC), and multiclass classification (MC) tasks, with the regression task further assessed for its ability to facilitate classification-based predictions for the training and validation sets.

As the majority of the models developed showed high performance values on the training sets (Supplementary Tables S2a, S3a and S4a), we evaluated the performance using five-fold cross-validation on training sets in order to select the best models. More precisely, models characterized by the highest mean Q^2^_F3_, BA, and macro-BA for, respectively, regression, binary classification, and multiclass classification (for internal validation), were selected. Ultimately, the R-CatBoost, BC-CatBoost, and MC-CatBoost models were retained as the best models since they were characterized by the highest Q^2^_F3_, BA, and macro-BA (0.34 ± 0.05, 0.74 ± 0.02, 0.69 ± 0.02, respectively) (Table 2).

For the validation set, the R-CatBoost regression algorithm achieved an R^2^ of 0.43 and a Q^2^_F3_ of 0.39 (Table 2, Supplementary Table S2a). Furthermore, the mean absolute error (MAE) is reported at 20.09 (F%) within the range of 0 to 100 (F%). The RMSE is also significant, with a value of 25.86 (F%). Absolute F% errors of 10 and 20 are illustrated in Supplementary Figure S3. According to Wang et al., the RMSE of experimental measurements of oral bioavailability is 14.5 (F%) [33], which might explain this high RMSE.

We categorized the outcome predictions from the developed R-CatBoost model into two classes: high (greater than 50%) and low (less than 50%) oral bioavailability. We then evaluated the performance for binary classification using the 50% threshold, resulting in a BA of 0.77 (Table 2, Supplementary Table S3a). In comparison, the best model trained on binary data, where values are dichotomized into 1 (greater than 50%) and 0 (less than 50%), showed a lower BA, with the BC-CatBoost classification method achieving a BA of 0.74 (Supplementary Table S3a).

We applied the same processing for multiclass classification; we categorized outcome predictions from R-CatBoost regression into three classes: low (less than 30%), medium (higher than 30% and less than 60%), and high (higher than 60%) oral bioavailability. We then evaluated the performance for multiclass classification using the 30% and 60% thresholds, resulting in a lower macro-BA of 0.67 compared to the multiclass model, MC-CatBoost, which achieved a macro-BA of 0.70. The analysis of predictive performance under the 30–60% thresholds (Table 2, Supplementary Table S4a) further highlights notable trends and disparities among various machine learning approaches in multiclass prediction. On the validation set, the MC-CatBoost model trained for multiclass prediction achieved a BA of 0.77 for the <30% class and of 0.75 for the >60% class. However, these models had low reliability when predicting the intermediate class (between 30% and 60%), showing a lower BA of 0.57, alongside a pronounced inability to accurately identify chemicals in this range, exemplified by an SE of 0.25.

The R-CatBoost regression model, while exhibiting lower performance compared to the double-threshold MC-CatBoost classification model, offers superior versatility and effectiveness in predicting medium-F% chemicals. It achieved a BA of 0.60 and an SE of 0.58 for the medium-F% class, demonstrating its utility in addressing the complexities of multiclass prediction tasks. These results emphasize the importance of methodology selection, with regression models proving particularly advantageous for medium-class prediction.

#### 2.2.2. Volume of Distribution Performance

Multiple ML models were trained and evaluated for their robustness in predicting VD_ss_. Regression models were evaluated for the prediction of continuous values and for dichotomous and multiclass predictions with 1 L·kg^−1^ and 0.6 L·kg^−1^–5 L·kg^−1^ categorization thresholds. The models were evaluated using dedicated metrics.

Molecular descriptor selection utilizing the VSURF algorithm on Mordred molecular descriptors yielded a subset of 26 molecular descriptors for a Topliss ratio (number of training chemicals per molecular descriptor) of 45:1, in compliance with the recommended threshold (>5) to avoid overfitting. These selected molecular descriptors were used as input to train the CatBoost, XGBoost, and RF models.

In order to select the best models, we only considered the performance obtained in five-fold cross-validation on training sets. More precisely, the best models corresponded to those characterized by the lowest mean GMFE, the highest BA, and the highest macro-BA for, respectively, the regression, binary classification, and multiclass models (for internal validation). According to this logic, the R-RF, BC-Chemprop, and MC-Chemprop models were retained as the best models since they were characterized by the lowest GMFE, highest BA, and macro-BA (2.19 ± 0.08, 0.78 ± 0.02, and 0.73 ± 0.02, respectively) (Supplementary Tables S5b, S6b and S7b). These models performed well on training data, with a GMFE below 2 (Supplementary Tables S5a, S6a and S7a).

For validation set 1, the R-RF regression algorithm achieved a GMFE of 2.35 (Supplementary Table S4a), indicating that the model can be regarded as sufficiently precise [34]. The R-RF regression model was able to predict VD_ss_ values mostly within 2-fold to 3-fold errors (Supplementary Figure S4).

From the developed R-RF model, we categorized the outcome predictions into two classes: high (greater than 1 L·kg^−1^) and low (less than 1 L·kg^−1^) VD_ss_. We then evaluated the performance for binary classification using the 1 L·kg^−1^ threshold, resulting in a BA of 0.75, showing comparable performance to that of the best model, BC-Chemprop trained on binary data, where values are dichotomized into 1 (greater than 1 L·kg^−1^) and 0 (less than 1 L·kg^−1^), which achieved a BA of 0.76 (Supplementary Table S6a).

We applied the same processing for multiclass classification; we categorized the outcome predictions from R-RF regression into three classes: low (less than 0.6 L·kg^−1^), medium (higher than 0.6 L·kg^−1^ and less than 5 L·kg^−1^), and high (higher than 5 L·kg^−1^) VD_ss_. We then evaluated the performance for multiclass classification using the 0.6 L·kg^−1^ and 5 L·kg^−1^ thresholds, resulting in a lower macro-BA of 0.68 (Table 3) compared to the best-performing multiclass model, MC-Chemprop, which achieved a macro-BA of 0.72. The analysis of predictive performance under the 0.6–5 L·kg^−1^ threshold (Table 3) did not reveal notable trends, as all classes had a BA greater than 0.60 for all algorithms (Supplementary Table S7a).

The R-RF model AD was further explored, and the model was used to map EDCs.

### 2.3. Applicability Domain

#### 2.3.1. Oral Bioavailability Applicability Domain

**Figure 2:**
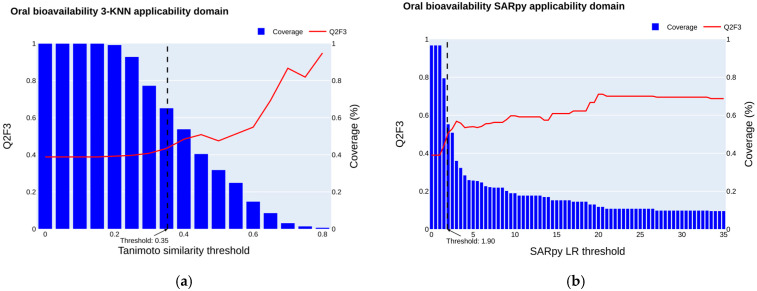
The effect of different definitions of applicability domains on coverage and predictive performance. (**a**) The 3-NN Tanimoto AD. The evolution of Q^2^_F3_ performance between observed and predicted values (red) on the validation set, between predicted and real values according to different Tanimoto thresholds ranging from 0 to 1. The evolution of the validation set coverage is plotted as blue bars. The model tested is the CatBoost regression method predicting F% values. (**b**) The SARpy LR AD. The evolution of Q^2^_F3_ performance (red) on the validation set according to different Log ratio thresholds relative to the structural alert associated with query chemicals. The evolution of the validation set coverage is plotted as blue bars.

The applicability domain of the regression model (R-CatBoost was assessed according to the best mean Q^2^_F3_ of the 50 iterations of 5f-CV) was assessed using a three-nearest neighbor approach on the validation set. The same plot reports the Q^2^_F3_ performance and the coverage according to different Tanimoto thresholds for the R-CatBoost regression models (Figure 2a). This model showed good overall performance in predicting oral bioavailability for low, medium, and high categories. As the applicability domain narrows, the validation set comprises more structurally similar compounds to the training set, and our models exhibit enhanced performance.

This improvement stems from the models’ ability to effectively recognize and learn the inherent patterns within the data. However, at higher threshold levels, occasional declines in R^2^ performance are observed. These fluctuations arise due to certain compounds being inaccurately predicted despite their structural resemblance to those in the training set. Additionally, as the number of compounds used for performance evaluation decreases, the uncertainty in performance metrics increases. Assessing performance based on a small dataset introduces variability, which can compromise the reliability and robustness of the models. While restricting the applicability domain can enhance performance, it is crucial to maintain a balance between predictive accuracy and the number of retained compounds to ensure the validity of the models. Here, we considered a minimum coverage of 60% of chemicals in the validation set retained, corresponding to a Q^2^_F3_ of 0.46.

Finally, we considered a Tanimoto threshold of 0.35 when applying the threshold formula Dc = <y> + Z × sigma, with <y> equal to 0.35, Z equal to 0.5, and a sigma of 0.14. This threshold resulted in a Q^2^_F3_ improvement from 0.39 to 0.43 and an MAE decrease from 20.09 to 18.9 with a coverage of 65%.

We explored the use of the Log ratio (LR) given by the MC-SARpy multiclass model to define the applicability domain. We plotted Q^2^_F3_ by varying the LR threshold from 0 to the maximum values of LR (infinite values transformed to maximum LR) alongside the size of the retained validation set (Figure 2b). Q^2^_F3_ increases as the thresholds are raised. Structural fragments defined by the MC-SARpy model (Supplementary Table S8) can be employed to provide insights into the reliability of predictions and identify significant structural features that move chemicals toward either high or low F% values.

When we use SARpy to define the applicability domain and consider a threshold corresponding to a coverage of 65% (LR of 1.90), as we did when analyzing the applicability domain defined by the k-nearest neighbor approach, we obtain a Q^2^_F3_ of 0.46. This predictive performance is slightly better than that obtained with the k-nearest neighbor method and can be used as the applicability domain definition to improve the model’s performance. Both approaches can be used together to define the AD, each providing deeper insight into the prediction.

#### 2.3.2. Volume of Distribution Applicability Domain

**Figure 3:**
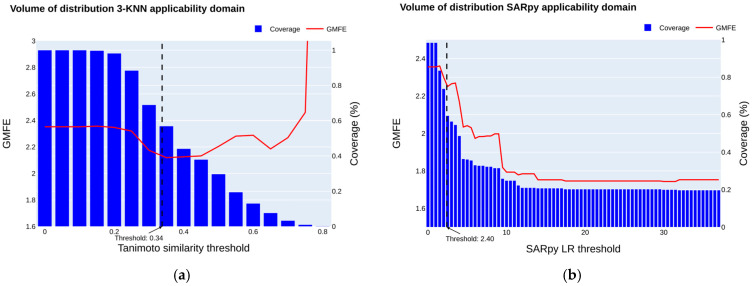
The effect of different definitions of applicability domains on coverage of validation set 1 and predictive performance (**a**) The 3-NN Tanimoto AD. The evolution of the GMFE (in red) on validation set 1 according to different Tanimoto thresholds ranging from 0 to 1. The evolution of the coverage of the validation set is plotted as blue bars. (**b**) The SARpy AD. The evolution of the GMFE performance (in red) on the validation set according to different Log ratio thresholds relative to the structural alert associated with the prediction. The evolution of the effective size retained in the validation set is plotted alongside in blue. The plot was made using the SARpy model with the 0.6–5 L·kg^−1^ threshold.

The applicability domain of the best regression model (R-RF was assessed according to the best mean GMFE of the 50 iterations of 5f-CV) was explored using a three-nearest neighbor approach on the validation set. In the same plot, the GMFE performance and the coverage of chemicals retained according to varying Tanimoto thresholds for the R-RF regression model are illustrated in Figure 3a.

As the threshold is further increased, the GMFE stops decreasing and starts increasing, as was seen in the QSAR model for oral bioavailability. We considered a minimum coverage of 60% of chemicals in the validation set retained.

Finally, we considered a Tanimoto threshold of 0.34 when applying the threshold formula Dc = <y> − Z × sigma, with <y> equal to 0.42, Z equal to 0.5, and a sigma of 156. This threshold improved the GMFE from 2.35 to 2.17, becoming closer to 2, with a coverage of 61%.

We used the Log ratio (LR) given by the MC-SARpy multiclass model as an applicability domain definition. We plotted the GMFE by varying the LR threshold from 0 to the maximum values of LR (infinite values transformed to maximum LR), alongside the effective data retained in the validation set (Figure 3b).

The GMFE decreases as the thresholds are increased. When a threshold corresponding to a coverage of 63% is considered (LR of 2.40), similarly to what is described for the k-nearest neighbor approach, a GMFE equal to 2.24 is observed. SARpy structural fragments defined by the MC-SARpy model can be used (Supplementary Table S9) to provide insights into the reliability of predictions and identify significant structural features that modulate chemical activity toward either high or low VD_ss_ values.

### 2.4. Molecular Descriptor Importance

**Figure 4:**
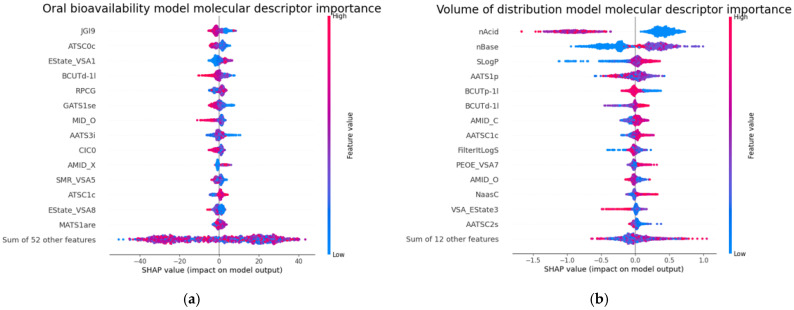
(**a**) A summary plot obtained using the SHAP package. The plot shows the importance of the 15 most important molecular descriptors of the R-CatBoost (regression) oral bioavailability model and their effects on predictions. The plot depicts the relationship between a molecular descriptor’s value and its impact on the prediction. For instance, high values of jGI9 (a topological charge molecular descriptor) are associated with a tendency to decrease oral bioavailability. (**b**) A summary plot obtained using the SHAP package for the R-RF (regression) VD_ss_ model. The plot shows the importance of the 15 most important molecular descriptors and their effect on the predictions. This plot depicts the relationship between a molecular descriptor value and its impact on the prediction. For example, high values of SLogP tend to increase VD_ss_.

The importance of the molecular descriptors in the best models was analyzed using the SHAP (SHapley Additive exPlanations) values with the SHAP Python package (version 0.44.0) [35]. The SHAP value for each molecular descriptor (in rows) indicates the degree to which a model’s computed predictions change when the values of molecular descriptors vary. In Figure 4, all the SHAP values for the top 15 molecular descriptors are displayed in rows. The x-axis represents the SHAP values, while the y-axis depicts the molecular descriptors, ordered by importance from highest (at the top) to lowest (at the bottom). Each dot corresponds to a chemical and is color-coded according to the value of the corresponding molecular descriptor, ranging from high to low.

Among the top 15 most important molecular descriptors for the R-CatBoost regression oral bioavailability model (Figure 4a), we observe complex molecular descriptors that retain topological and electrostatic information, with the JGI9 (9-ordered mean topological charge), ATSC0c (centered Moreau–Broto autocorrelation of lag 0 weighted by Gasteiger charge), Estate_VSA1 (Labute’s Approximate Surface Area EState indices and surface area), BCUTd-1I (first lowest eigenvalue of Burden matrix weighted by sigma electrons), and MID_O (molecular ID on O atoms) molecular descriptors being of most importance in the model.

These molecular descriptors are consistent with those identified in previous models developed for oral bioavailability. For instance, the model by Wei et al. [9] highlighted SsOH (an E-state molecular descriptor), ATS5i (a topological structure molecular descriptor), and TopoPSA(NO) as the most important. Similarly, the model by Ma et al. [16] identified additional topological structure descriptors, including TopoPSA and TopoPSA(NO), along with an E-state molecular descriptor (EState_VSA8) and the MID_O molecular descriptor, which is related to the identification and characterization of oxygen atoms in chemicals.

For VD_ss_, Figure 4b depicts the molecular descriptor importance for the R-RF regression model. Among the top 15 molecular descriptors, we observe those that impact the model’s prediction. We observe that low numbers of acidic groups increase VD_ss_ and low numbers of base groups decrease VD_ss_. Another important molecular descriptor is the logarithm of the n-octanol–water partition coefficient (SLogP), an important factor in pharmacokinetics. These molecular descriptors were previously found to impact VD_ss_ [23].

The list of molecular descriptors, along with their Mordred molecular descriptions and examples of chemicals with high and low values, is provided in Supplementary Tables S10 and S11.

### 2.5. QSAR Mapping of EDCs as a Function of Key TK Properties

In order to characterize the TK profiles of chemicals regarded as endocrine disruptors (EDCs), we predicted key TK properties for 131 EDCs by applying three QSAR models: the two QSAR models for oral bioavailability and VD_ss_ described in this manuscript, using the SARpy AD, and an existing QSAR (from VEGA) model predicting the total body elimination half-life, for which we considered moderate and good experimental predictions to be inside the AD.

**Figure 5:**
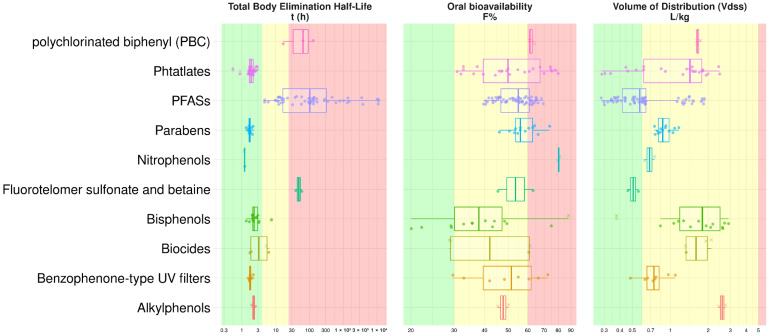
Boxplots of the predictions for oral bioavailability, VD_ss_, and elimination half-life for a set of 131 EDCs categorized into 10 chemical categories. Perfluoroalkylcarboxylic acid (PFCA), perfluoroalkylsulfonic acid (PFSA), perfluoroalkylether acid (PFEA), perfluoroalkane sulfonamide derivative (PASF), perfluoroalkyl phosphonic acid (PFPiA), and polyfluoroalkyl phosphate diester (diPAP) categories were grouped as PFASs. Chemicals inside the SARpy AD are represented as circles, and chemicals outside as crosses. Background colors are set to green, orange, and red for, respectively, low, medium, and high values of VD_ss_ (thresholds: 0.6 L·kg^−1^ and 5 L·kg^−1^), oral bioavailability (thresholds: 30% and 60%), and elimination half-life (thresholds: 4 h and 24 h).

The oral bioavailability and volume-of-distribution prediction results for the targeted EDCs (categorized into 10 common chemical families) are shown in Figure 5, in addition to the total body elimination half-life prediction.

Among the studied chemical categories, perfluoro (alkyl/alkane) substances (PFASs) exhibited a long total body elimination half-life, suggesting prolonged retention in the body. However, these compounds typically had a low VD_ss_, with the exception of PFASFs (Supplementary Figure S5), which demonstrated a moderate predicted VD_ss_. Bisphenols, on the other hand, exhibited a moderate VD_ss_, indicating a balanced distribution across tissues, and displayed medium oral bioavailability. These compounds were characterized by a relatively short elimination half-life, implying faster clearance from the body compared to PFASs.

**Figure 6:**
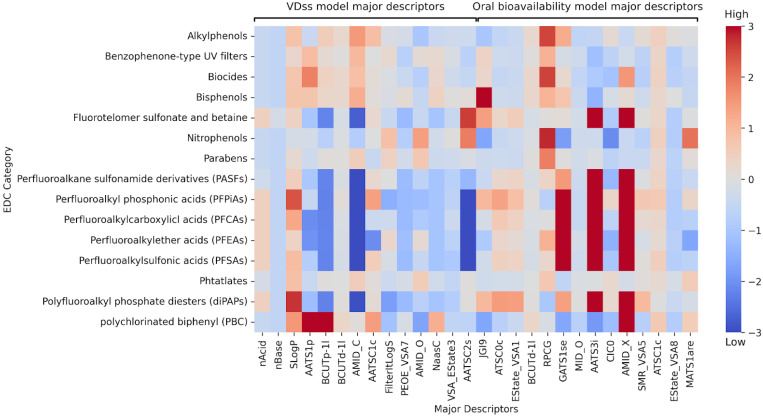
Heatmap of the mean standardized descriptor values for the EDC list, grouped by EDC families, based on the major descriptors of the oral bioavailability and VD_ss_ models. Standardization was performed using the mean and standard deviation computed from the training set.

To assess the molecular descriptors driving model predictions, we visualized a heatmap of the mean standardized descriptor values for EDC compounds, grouped by chemical family, based on the major descriptors used in the oral bioavailability and VD_ss_ models (Figure 6). The results reveal that PBCs and nitrophenols exhibit notably low values of GATS1se and AATS3i, features associated with high predicted oral bioavailability in the model. These characteristics could contribute to the model assigning elevated oral bioavailability to compounds in these families. PFASs display low values of BCUTp-1l, AMID_C, and AATSC2s, combined with high nAcid and low nBase counts. The model predicted a low volume of distribution (VD_ss_) for these chemical families, suggesting that these structural traits are key drivers of the pharmacokinetic behavior predicted for these EDCs.

Interestingly, only a few chemicals were inside the AD of the VD_ss_ and oral bioavailability models, with, respectively, 16 and 27 chemicals inside the 3-NN Tanimoto AD. For the elimination half-life, where a good prediction is considered to be inside the applicability domain, 24 chemicals were considered. Among them, Phthalates (DBP, DCHP, DEP, DMP, …), Benzophenone-type UV filters, 4-n-Nonylphenol (Alkylphenols), and Benzylparaben emerged for the VD_ss_ model, and Phthalates (DBP, DCHP, DEP, DMP, …), 4-n-Nonylphenol (Alkylphenols), Benzophenone-type UV filters, Parabens, and Bisphenols (BPE, BPF, BPS) for the oral bioavailability model.

**Figure 7:**
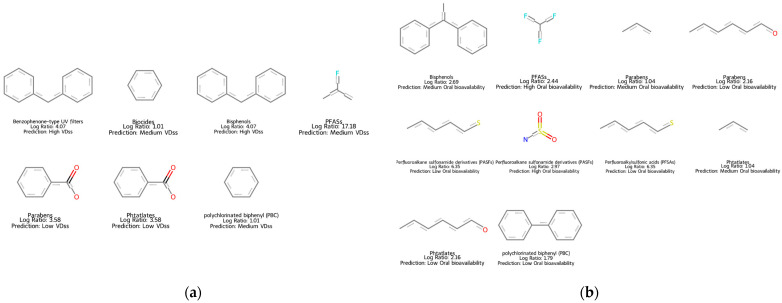
Structural fragment alerts identified by MC-SARpy in more than two chemicals across different EDC categories for the VD_ss_ (**a**) and oral bioavailability (**b**) models. The Log ratio and the predicted category associated with each structural alert are reported. PASFs, PFCAs, PFEAs, PFSAs, diPAPs, and PFPiAs were combined into the PFAS category when the structural fragment was identical.

With the SARpy AD, of 131 chemicals, 118 and 91 were inside the AD for VD_ss_ and oral bioavailability, respectively. The method also allowed us to identify structural patterns among the groups of chemicals that were linked to high or low values of VD_ss_ or oral bioavailability. For example, aromatic rings or two aromatic rings linked, which are found in bisphenols, PBC, and benzophenone-type UV filters, are associated with medium or high values of VD_ss_. An aromatic ring linked to a carboxylic group, found in parabens and phthalates, is associated with low values of VD_ss_ (Figure 7a). Perfluoroalkyl groups, found in PFASs, are associated with high oral bioavailability, and long carbon chains, found in parabens, are associated with low oral bioavailability (Figure 7b).

In order to have an idea about the relevance of our predictive models for EDCs, we searched the literature for toxicokinetic (TK) profiles reported in humans. We found that TK profiles of bisphenols were assessed in piglets in the study by Gély et al. [36]. BPA and its alternatives exhibited low oral bioavailability, medium to high VD_ss_, and a short elimination half-life. Studies on humans have estimated the elimination half-life of deuterated BPA to be approximately 6.4 ± 2.0 h [37].

For benzophenone UV filters, the literature reports a short elimination half-life of around 4 h [38]. Per- and polyfluoroalkyl substances (PFASs) generally exhibit high oral bioavailability. For example, PFOA and EOF showed bioavailability values of 65–71% and 74–87%, respectively, in mouse studies [39]. In workers exposed to perfluoroalkyl surfactants, a low mean distribution volume of 0.08 L·kg^−1^ was reported [40]. Drew et al. [41] investigated the elimination half-lives of several PFASs, including PFOS, PFHpS, PFHxS, PFNA, and PFDA, reporting prolonged elimination half-lives of 74.1 ± 13.4 h, 45.7 ± 9.4 h, 9.3 ± 1.3 h, 12.3 ± 3.2 h, and 60.4 ± 10.4 h, respectively. Phthalates were found to have short elimination half-lives. For example, DEHP exhibited an elimination half-life of 4.3–6.6 h in humans [42]. Overall, these findings from the literature align with our TK QSAR models.

We also predicted TK properties for a set of 316 chemicals that are likely to disrupt ARs and ERs. Using dedicated thresholds for each TK property (VD_ss_: 0.6 kg/L and 5 kg/L; oral bioavailability: 30% and 60%; elimination half-life: 4 h and 24 h), we set chemical attributes as low, medium, and high TK concern to highlight chemicals that are characterized by concerning TK profiles in terms of chemical risk. Among the 316 chemicals, 67.4% (213 chemicals) were inside the SARpy AD of the oral bioavailability QSAR, 70.9% (224 chemicals) were inside the SARpy AD of the VD_ss_ QSAR, and 94.3% (298 chemicals) were inside the ADI of the elimination half-life QSAR.

Among the 316 chemicals, 16.4% (52 chemicals) were predicted as having a TK risk, with at least one TK property classified as high.

Among them, Bisphenol AF was predicted to have high oral bioavailability, a medium half-life, and a medium VD_ss_. This chemical poses a risk, as it is produced in large amounts—100 to 1000 tons, as stated by the European Chemical Agency (ECHA) [43]. Seven other chemicals were found to be registered in ECHA (4′,5′-Diiodofluorescein; 3,5-Dichloro-4-hydroxybenzophenone; 3-[1-[4-[2-(dimethylamino)ethoxy]phenyl]-2-phenylbut-1-enyl]phenol; 3′,6′-dihydroxyspiro [2-benzofuran-3,9′-xanthene]-1-one; 4′,5′-dibromo-3′,6′-dihydroxyspiro[isobenzofuran-1(3H),9′-[9H]xanthene]3-one; Bisphenol AF; mitotane; and 1-chloro-2-[2,2,2-trichloro-1-(4-chlorophenyl)ethyl]benzene). For example, the use of 4′,5′-Diiodofluorescein in cosmetic products was banned in Europe, 3-[1-[4-[2-(dimethylamino)ethoxy]phenyl]-2-phenylbut-1-enyl]phenol, and bisphenol AF were recognized as toxic to reproduction, and CLP describes 2,2,2,o,p′-pentachloroethylidenebisbenzene as fatal if inhaled and toxic if swallowed.

From the set of identified EDCs, three have a high TK risk, namely, (E,Z)-Tamoxifene, Clomiphene, and (E)-Toremifene, all having a high VD_ss_, high oral bioavailability, and a medium body elimination half-life. These chemicals are known to be related to endocrine disruption: Clomiphene is a drug that increases the chance of pregnancy by facilitating ovulation [44], Tamoxifen is a drug used to treat hormone-positive breast cancer [45], and Toremifene is known to bind to estrogen receptors and act as a weak partial agonist and potent antagonist [46]. Overall, these results show the relevance of using our QSAR models to predict EDCs and TK properties to identify chemicals that are most likely to pose a risk.

## 3. Discussion

This study used a large dataset comprising over 1600 chemicals to develop a QSAR model for both oral bioavailability and VD_ss_ for regression, binary classification, and multiclass prediction.

Among similar studies considering oral bioavailability at a 50% threshold, Falcón-Cano et al. (2020) [10] employed a dataset of over 1400 compounds and achieved a BA of 0.78. Venkatraman (2021) [11] used 1800 chemicals and reported a BA of 0.71, and Wei et al. (2022) [9] achieved an accuracy of 0.79. Our model exhibited comparable results to these studies, with a BA of 0.77, corresponding to an accuracy of 0.77 on a different validation set. Recently, Ma et al. (2024) [16] reported an accuracy of 0.82 using the same 209-compound validation set as Falcón-Cano et al. [10].

The QSAR model described in this article can be regarded as more robust than those previously published. In particular, our model was trained and evaluated on a larger dataset, with twice as many chemicals in our validation set compared to those used by Falcón-Cano et al., Wei et al., and Ma et al. (405 in our study vs. 209) [10,11,16].

For VD_ss_ model development in related work with similar numbers of substances that used the GMFE metric to evaluate their models, Lombardo et al. (2021) [17,18], who had fewer compounds in the training set and used the same validation set of 34 compounds, reported a GMFE of 1.70, while our regression model exhibited a GMFE of 1.81 (Supplementary Table S4a).

Our results are therefore comparable to those of previously published models and, as discussed for bioavailability, can be considered more robust given the larger training set size. In addition, we were able to model and compare the development of regression, binary classification, and multiclass classification models for this endpoint. The development of an applicability domain to determine the limit of our QSAR models and the application of SARpy resulted in some structural fragment alerts on EDCs that were linked to high or low values of VD_ss_ or oral bioavailability.

The development of our QSAR models followed the OECD QSAR validation principles. In line with Principle 1, the models have defined endpoints for oral bioavailability and VD_ss_. Principle 2 is addressed with an unambiguous algorithm (scripts available as Supporting Material together with model reporting formats, QMRF), defined methods, and the retained models—R-CatBoost, BC-CatBoost, and MC-CatBoost—for the prediction of oral bioavailability, corresponding to continuous value prediction, binary classification prediction, and multiclass prediction, respectively. For VD_ss_, the retained models are R-RF, BC-Chemprop, and MC-Chemprop, corresponding to continuous value prediction, binary classification prediction, and multiclass prediction, respectively. Principle 3 is addressed by an applicability domain, defined using two approaches: a structural alert approach using SARpy and an analog-based approach using a three-nearest neighbor method.

The models follow Principle 4 by ensuring appropriate measures of goodness of fit, robustness, and predictivity. This was demonstrated by strong performance on the training set (seen data) and the external validation set (unseen data), as well as through 50 iterations of five-fold cross-validation.

Principle 5, which concerns the definition of a mechanistic interpretation, is explored through model molecular descriptor importance and the applicability domain. The applicability domain both explores the nearest neighbors and allows identification of the most important structural fragments contributing to predictions with the SARpy models. Both approaches can be used together to define the AD, each providing deeper insight into the prediction. However, we recommend using the SARpy LR AD approach, as it offers a clearer understanding of the structural fragments responsible for the activity. Another definition of the applicability domain was explored using Insubria plots, relying on the leverage approach from the hat matrix [47]. Similarly to 3-NN, we can observe that around 95% of the compounds fall inside the applicability domain for oral bioavailability and VD_ss_ (Supplementary Figure S6).

## 4. Materials and Methods

### 4.1. Data

#### 4.1.1. Oral Bioavailability Data Source

Data on human oral bioavailability were collected from multiple sources, including OCHEM [48], CHEMBL [49], and articles by Min Wei et.al [9], Falcón-Cano et al. [10], Varma et al. [50], and Tian et al. [13]. In total, 1712 chemicals and associated oral bioavailability data were retrieved and curated. Special attention was paid to the presence of qualifiers (greater or less than a certain threshold) for oral bioavailability in order to properly consider this information with respect to the categorization thresholds adopted during the discretization of continuous values.

#### 4.1.2. Volume of Distribution Data Source

Data on human VD_ss_ values were collected from CHEMBL [49], the article by Lombardo et al. [18], and the article by Liu et al. [22]. In total, 1591 chemicals and associated VD_ss_ data were retrieved and curated.

#### 4.1.3. Preprocessing Standardization

All the chemicals were mapped to their PubChem Compound ID (CID) in order to harmonize chemical structures (SMILES) that are standardized according to the PubChem protocol [51] (i.e., normalization of the representation, implicit hydrogens, atom valence, tautomeric form representation, etc.). The PubChem CID was retrieved according to the available SMILES, CAS RN, name, and InChI available from the source database. In cases of chemicals with ions, the largest fragment was considered. This standardization allowed us to identify duplicate chemicals for which we computed the mean F% and mean VD_ss_ values. Duplicate chemicals with a difference greater than 20 for the standard deviation of F%, as well as those with F% values exceeding 100 or falling below 0, were excluded from the dataset.

### 4.2. Dataset Preparation for Modeling

#### 4.2.1. Oral Bioavailability Data Preparation

The datasets were randomly split into training and validation sets. To construct the validation set, chemicals were sorted based on their F%, and we selected every fourth chemical to populate this set. This choice ensured representative inclusion across the range of F% values. This approach resulted in ~25% of chemicals (405 chemicals) being selected for the validation set.

The remaining 75% of chemicals of the training set with known F% values were retained for regression modeling (1213 chemicals). Some chemicals in the literature did not have F% values but only qualitative information about low or high F% with respect to different thresholds (50%, 30%, and 60%). For the implementation of QSAR classifiers, chemicals with known threshold values were considered. This encompassed the design of a training set for binary classification (50% threshold, distinguishing low and high classes with 1307 chemicals) and another training set for multiclass classification (30–60% thresholds, defining low, medium, and high classes with 1244 chemicals). The 50% thresholds (single-threshold classification models) were used as described in the literature [9,10], and the 30–60% thresholds (double-threshold multiclass models) were used considering the thresholds adopted by some CRO experts in this domain (personal communication). No chemicals from the respective training set were included in the validation set.

#### 4.2.2. Volume of Distribution Data Preparation

The datasets for volume of distribution were randomly split into training and validation sets, similarly to the oral bioavailability data. This resulted in ~25% of chemicals being selected for the validation set (390 chemicals). A second validation set was developed using a list of 34 chemicals extracted from the article by Lombardo et al. (2016) [17]. This dataset, which did not contain chemicals belonging to sets used with previously published models, was used for model comparison. No chemicals from the two validation sets were included in the training set. Here, the natural logarithm (ln(VD_ss_)) was used to facilitate QSAR model development [20].

The classification model was developed on the training set (1167 chemicals), with a threshold of 1 L·kg^−1^ corresponding to a chemical extensively (90%) distributed in tissues [52]. For multiclass classification, we considered thresholds of 0.6 L·kg^−1^ and 5 L·kg^−1^ [7].

### 4.3. Molecular Descriptors

We computed Mordred molecular descriptors [53] covering a wide range of structural and physico-chemical properties of chemicals. A total of 1826 molecular descriptors were computed. Subsequently, columns containing “NA” values, molecular descriptors with zero variance, and those exhibiting absolute pair correlations exceeding 0.97 were excluded from the dataset to reduce redundant information. This process yielded 560 molecular descriptors for the oral bioavailability regression dataset, 507 for the binary classification dataset, 560 for the multiclass classification dataset, and 500 for the volume-of-distribution datasets.

### 4.4. Selection of Molecular Descriptors

In order to reduce the number of molecular descriptors (i.e., the independent variables of the models) and increase the parsimony and interpretability of the models, the VSURF algorithm [54] was applied to select and retain only the most informative molecular descriptors. The R package VSURF (version 1.2.0) allows identification of the most informative molecular descriptors using random forest importance scores based on permutation and using a stepwise forward strategy that selects the variables of the most accurate models. Molecular descriptor selection was performed exclusively on the training sets to avoid data leakage. VSURF identifies two sets of molecular descriptors: interpretation and prediction levels. We selected the interpretation set, as it contains the most molecular descriptors. We measured the Topliss ratio as the number of training chemicals per molecular descriptor, in accordance with OECD guidelines, which recommend a rule-of-thumb ratio greater than 5 [55].

### 4.5. Machine Learning Algorithms

We considered a variety of machine learning models, such as CatBoost [56], XGBoost [57], random forest (RF) [58], and Chemprop [59]. The SARpy [60,61] method, a structure–activity relationship (SAR) method, was also tested for prediction to enhance the mechanistic interpretability of QSAR models. A description of each machine learning approach is available in Supplementary Materials (References [62,63,64] are cited in the Supplementary Materials). CatBoost, XGBoost, chemprop, and RF were applied to regression, binary classification, and multiclass classification. SARpy was used for binary and multiclass classification.

### 4.6. Protocol

The tuning of algorithm hyperparameters was optimized using the training set without using any data from the validation sets.

For CATBoost, XGBoost, and RF, 5-fold cross-validation was carried out for hyperparameter optimization using a grid search (Supplementary Table S1).

After this step, the models were subjected to 50 iterations of 5-fold cross-validation using the training set in order to assess algorithm robustness and identify the best-performing algorithm. Specifically, the training set was partitioned into five non-overlapping subsets 50 times. In each iteration, four subsets were combined to train the model, while the remaining subset was used to evaluate model performance in cross-validation.

The predictive performance of the “unseen” chemicals in the validation datasets was then assessed according to commonly used statistical indicators, i.e., sensitivity (SE), specificity (SP), and balanced accuracy (BA) for the binary classification; class-specific SE, SP, and BA, macro/micro-SE, macro/micro-SP, and macro/micro-Ba for multiclass classification; Root Mean Squared Error (RMSE), mean absolute error (MAE), Q^2^_F3_, and R^2^ for oral bioavailability regression models; and geometric mean fold error (GMFE) for VD_ss_ models. The regression model with the best performance on the external validation set was also evaluated for its performance in binary and multiclass classification tasks (Supplementary Figure S2).

### 4.7. Predictive Performance Metrics

The models were evaluated externally and internally using their respective training and validation sets, whose data were not used to calibrate or optimize the models.

The performance metrics adopted for classification are defined as follows:

(1) $$\mathrm{Sensitivity}\;(\mathrm{SE})\;=\;\frac{TP}{TP\;+\;FN}$$

(2) $$\mathrm{Specificity}\;(\mathrm{SP})=\frac{TN}{TN+FP}$$

(3) $$\mathrm{Balanced}\;\mathrm{accuracy}\;(\mathrm{BA})=\frac{Sensitivity+Specificity}{2}$$

where *TP* stands for True Positive, *TN* for True Negative, *FN* for False Negative, and *FP* for False Positive. For multiclass prediction, these same metrics were computed for each category at a time by considering the category under scrutiny as active.

For multiclass classification models, we evaluated performance using metrics calculated individually for each class by treating it as the positive class, providing class-specific SE, SP, and BA. Additionally, we computed micro-averaged metrics, which aggregate all instances to give equal weight to each sample, and macro-averaged metrics, which average performance across classes equally, ensuring balance between majority and minority classes. This combination offers both detailed class-level insights and an overall performance assessment.

For the oral bioavailability regression models, the performance metrics were defined as follows [65]:

(4) $$RMSE=\sqrt{\frac{\sum (yi-\hat{y}i{)}^{2}}{n}}$$

(5) $$R^{2}\left(y,\hat{y}\right)=1-\frac{\sum_{i=1}^{n}{\left(yi-\hat{y}i\right)}^{2}}{\sum_{i=1}^{n}{\left(yi-\underline{y}\right)}^{2}}$$

(6) $$Q_{F3}^{2}\left(y,\hat{y}\right)=1-\frac{\left(\sum_{i=1}^{n\left(OUT\right)}{\left(y_{i}-{\hat{y}}_{i}\right)}^{2}\right)/n_{OUT}}{\left(\sum_{i=1}^{n\left(TR\right)}{\left(y_{i}-{\hat{y}}_{TR}\right)}^{2}\right)/n_{TR}}$$

(7) $$MAE\left(y,\hat{y}\right)=\frac{1}{n_{samples}}\sum_{i=0}^{n_{samples}-1}\left|yi-\hat{y}i\right|$$

For the modeling of VD_ss_, we assessed the performance of the regression models using the geometric mean fold error (GMFE) [17,18].

The GMFE was computed as follows:

(8) $$GMFE\left(y,\hat{y}\right)=10^{\left(\sum_{i=1}^{n}\left|Log_{10}\left(\frac{\hat{y}}{yi}\right)\right|\right)/n}$$

where *ŷi* is the predicted value of the *i*-th sample, and *yi* is its corresponding experimental value; $\underline{y}$ is the mean of the predicted values. $n_{TR}$ and $n_{OUT}$ are the number of training and validation chemicals, respectively; ${\underline{y}}_{TR}$ is the average value of the training set experimental responses; and ${\underline{y}}_{OUT}$ is the average value of experimental responses of external validation chemical values.

The VD_ss_ regression models were trained using logarithmic values, and the linear predicted values were subsequently adopted for the GMFE formula. GMFE is a standard metric for evaluating PK models when the model is trained with values in the logarithmic space and the predicted values are recovered through y = e^y(log)^, where y(log) is the predicted value in the logarithmic space [66]. GMFE values around and below 2 are generally considered indicators of acceptable precision for pharmacokinetic parameters [34].

The predictive performance of the regression models was also evaluated in terms of classification. For this evaluation, the validation set was predicted with the best model and then classified according to the corresponding thresholds of oral bioavailability and VD_ss_.

### 4.8. Definition of the Applicability Domain

#### 4.8.1. K-Nearest Neighbors

In QSAR modeling, the applicability domain refers to the precision of the prediction computed by a model within a given chemical space, thereby providing information about the expected level of reliability of predictions computed for unseen chemicals included in the molecular descriptor space defined by the applicability domain (AD). The applicability domain helps prevent model misuse and enhances the trustworthiness of predictions. It is established by considering the training set and serves as a guideline for determining which chemicals the model can assess with a given reliability [67].

Various methods exist for defining the applicability domain in QSAR. In this study, a distance-based approach was chosen (3-NN Tanimoto AD), evaluating the similarity between a query chemical and those in the training set. This similarity was measured using the Tanimoto score, which measures chemical similarity using chemicals encoded as fingerprints; here, we used Morgan fingerprints [62]. For each query chemical in the validation set, we calculated the average Tanimoto score of the three most similar compounds from the training set. The models’ predictive performance was then characterized by analyzing the precision of predictions across different threshold values for the Tanimoto score.

Establishing a useful threshold to determine whether a chemical falls within the applicability domain requires balancing precision and coverage of the chemical space. Various methods exist for setting this threshold, and in this study, we defined it as Dc = <y> − Zσ. Here, ⟨y⟩ represents the average Tanimoto score of the three closest training set neighbors for each chemical, while σ\sigmaσ denotes the standard deviation of these scores. The parameter Z controls the significance level, with a default value of 0.5. Unlike the original formula, which uses Euclidean distance (where 0 signifies identical chemicals), our approach subtracts Zσ from ⟨y⟩, adapting it to the Tanimoto score scale (ranging from 0 to 1, where 1 indicates identical chemicals) [68,69].

#### 4.8.2. SARpy

We also investigated the possibility of applying SARpy to define the applicability domain (SARpy AD). For this purpose, we took into account the likelihood ratio (LR) for each structural alert (SA) associated with each predicted chemical to gauge its precision in correctly predicting the chemical. A chemical was considered to be within the applicability domain if the LR of the structural alert responsible for the predicted compound exceeded a specific threshold. The predictive performance of the models was then compared by evaluating their effectiveness across various LR threshold values.

### 4.9. Mapping of EDCs

We mapped the TK space of EDCs on two lists of chemicals, the first one containing a selection of 131 endocrine-disrupting chemicals reported in the literature [70]. These EDCs can be found in everyday life in different products, including additives, food packaging, food and beverage containers or cans, cosmetics, cookware, toys, hygiene and cleaning products, etc. [71,72,73]. Many of these chemicals can be found at detectable levels in the urine and blood of children and adults [74,75,76].

The second set, consisting of 55,450 chemicals to which humans are potentially exposed, forms a list of toxicological and environmental chemicals of interest [77]. From this set, we applied QSAR models for estrogen binding [78] and androgen binding [79] and selected the chemicals most likely to perturb the considered receptors by considering only QSAR predictions characterized by an applicability domain index greater than >0.8. This strict requirement resulted in a subset of 316 chemicals being retained for screening.

Oral bioavailability and VD_ss_ were computed by the QSAR models described herein. In addition, the elimination half-life was computed using the freely available dedicated VEGA QSAR [78] model, and the TK properties of different groups of EDCs were compared.

All the models and code for the prediction of oral bioavailability and volume of distribution at steady state are available at https://github.com/guillaumeolt/QSAR_TK (accessed on 16 September 2025).

## 5. Conclusions

In this study, we developed QSAR models to predict oral bioavailability and VD_ss_ using state-of-the-art machine learning approaches and following OECD guidelines. Leveraging current databases for these pharmacokinetic endpoints, we developed regression, binary classification, and multiclass classification models and systematically evaluated their performance across various scenarios using relevant metrics. We applied a 3-NN applicability domain approach and highlighted the importance of using SAR methods to define applicability domains. Furthermore, we integrated these QSAR models with a complementary elimination half-life model and applied them to a curated list of endocrine disruptors and a list of toxicological and environmental chemicals of interest. This combined approach identified critical categories and chemicals of concern, providing valuable insights for the prioritization and regulatory evaluation of endocrine-disrupting chemicals.

## Figures and Tables

**Table 1:** Number of chemicals used to develop QSAR models, according to the modeling algorithms, for oral bioavailability and VD_ss_.

Endpoint	Dataset	Modeling Algorithm	Number of Chemicals
Oral bioavailability	Training	Regression	1213
		Classification (50% threshold)	1307
		Binary classification (30% and 60% thresholds)	1244
	Validation	Regression/binary classification/multiclass classification	405
VD_ss_	Training	Regression/binary classification/multiclass classification	1167
	Validation 1		390
	Validation 2		34

**Table 2:** Performance obtained for the QSAR models in predicting oral bioavailability on the validation set. The predictive performance of the algorithms was evaluated across regression (R), binary classification (BC), and multiclass classification (MC) tasks. NA means not applicable.

Metric	Performance for Regression (R)	Performance for Binary Classification (BC)	Performance for Multiclass Classification (MC)	Cross-Validation (CV) Performance for Regression (R)	CV Performance for Binary Classification (BC)	CV Performance for Multiclass Classification (MC)
	Validation Set			CV		
Model	R-CatBoost	BC-CatBoost	MC-CatBoost	R-CatBoost	BC-CatBoost	MC-CatBoost
*Regression metrics*						
RMSE	25.86	NA		27.71 ± 0.98		
R^2^	0.42	NA	NA	0.38 ± 0.04	NA	NA
MAE	20.09	NA	NA	20.90 ± 0.82	NA	NA
MedAE	15.92	NA	NA	17.01 ± 1.11	NA	NA
Q^2^_F3_	0.39	NA	NA	0.34 ± 0.05	NA	NA
*Binary classification metrics*						
Sensitivity	0.78	0.79	NA	0.75 ± 0.03	0.78 ± 0.03	NA
Specificity	0.76	0.68	NA	0.72 ± 0.03	0.69 ± 0.04	NA
Balanced accuracy	0.77	0.74	NA	0.74 ± 0.02	0.74 ± 0.02	NA
*Multiclass classification metrics*						
Sensitivity (<30%)	0.46	NA	0.67	0.45 ± 0.05	NA	0.64 ± 0.05
Specificity (<30%)	0.91	NA	0.86	0.93 ± 0.03	NA	0.83 ± 0.03
Balanced accuracy (<30%)	0.68	NA	0.77	0.63 ± 0.02	NA	0.74 ± 0.02
Sensitivity [30–60%]	0.58	NA	0.25	0.69 ± 0.02	NA	0.31 ± 0.05
Specificity [30–60%]	0.63	NA	0.89	0.63 ± 0.03	NA	0.88 ± 0.02
Balanced accuracy [30–60%]	0.60	NA	0.57	0.63 ± 0.03	NA	0.60 ± 0.03
Sensitivity (>60%)	0.63	NA	0.83	0.63 ± 0.04	NA	0.79 ± 0.03
Specificity (>60%)	0.84	NA	0.67	0.84 ± 0.03	NA	0.70 ± 0.03
Balanced accuracy (>60%)	0.74	NA	0.75	0.74 ± 0.02	NA	0.74 ± 0.02
Macro sensitivity	0.56	NA	0.58	0.57 ± 0.03	NA	0.58 ± 0.02
Macro specificity	0.79	NA	0.81	0.80 ± 0.01	NA	0.81 ± 0.01
Macro balanced accuracy	0.68	NA	0.70	0.68 ± 0.02	NA	0.69 ± 0.02
Micro sensitivity	0.56	NA	0.64	0.57 ± 0.03	NA	0.63 ± 0.02
Micro specificity	0.78	NA	0.82	0.79 ± 0.01	NA	0.82 ± 0.01

**Table 3:** Predictive performance of the QSAR models for the prediction of VD_ss_ as a function of validation set 1. The predictive performance of the algorithms was evaluated across regression (R), binary classification (BC), and multiclass classification (MC) tasks.

Metric	Regression Model Performance	Classification Model Performance	Multiclass Classification Model Performance	CV Regression Model Performance	CV Classification Model Performance	CV Multiclass Classification Model Performance
	Validation Set 1			CV		
Model	R-RF	BC-Chemprop	MC-Chemprop	R-RF	BC-Chemprop	MC-Chemprop
*Regression metrics*						
GMFE	2.35	NA	NA	2.19 ± 0.08	NA	NA
*Binary Classification metrics*						
Sensitivity	0.79	0.77	NA	0.79 ± 0.03	0.73 ± 0.06	NA
Specificity	0.71	0.75	NA	0.75 ± 0.03	0.83 ± 0.04	NA
Balanced accuracy	0.75	0.76	NA	0.77 ± 0.02	0.78 ± 0.03	NA
*Multiclass classification metrics*						
Sensitivity (<0.6)	0.62	NA	0.68	0.66 ± 0.04	NA	0.71 ± 0.05
Specificity (<0.6)	0.91	NA	0.89	0.90 ± 0.02	NA	0.87 ± 0.03
Balanced accuracy (<0.6)	0.76	NA	0.78	0.78 ± 0.02	NA	0.79 ± 0.03
Sensitivity [0.6–5]	0.82	NA	0.76	0.83 ± 0.03	NA	0.76 ± 0.05
Specificity [0.6–5]	0.51	NA	0.63	0.57 ± 0.04	NA	0.66 ± 0.05
Balanced accuracy [0.6–5]	0.67	NA	0.70	0.70 ± 0.02	NA	0.71 ± 0.03
Sensitivity (>5)	0.22	NA	0.45	0.32 ± 0.06	NA	0.42 ± 0.09
Specificity (>5)	0.97	NA	0.94	0.97 ± 0.01	NA	0.94 ± 0.02
Balanced accuracy (>5)	0.60	NA	0.69	0.64 ± 0.03	NA	0.68 ± 0.04
Macro sensitivity	0.56	NA	0.63	0.60 ± 0.03	NA	0.63 ± 0.03
Macro specificity	0.80	NA	0.82	0.81 ± 0.01	NA	0.82 ± 0.02
Macro balanced accuracy	0.68	NA	0.72	0.71 ± 0.02	NA	0.73 ± 0.02
Micro sensitivity	0.65	NA	0.68	0.68 ± 0.02	NA	0.69 ± 0.03
Micro specificity	0.83	NA	0.84	0.84 ± 0.01	NA	0.84 ± 0.01
Micro balanced accuracy	0.74	NA	0.76	0.76 ± 0.02	NA	0.77 ± 0.02

## Data Availability

The original contributions presented in this study are included in the article/Supplementary Material. Further inquiries can be directed to the corresponding author.

## Supplementary Materials

The following supporting information can be downloaded at: https://www.mdpi.com/article/10.3390/jox15050166/s1, Table S1: Optimized parameters for the machine learning models, Table S2a: Performance table of the regression model of oral bioavailability, Table S2b: Cross validation performance table of the regression model of oral bioavailability, Table S3a: Performance table of the classification model of oral bioavailability, Table S3b: Cross validation performance table of the classification model of oral bioavailability, Table S4a: Performance table of the multiclass classification model of oral bioavailability, Table S4b: Cross validation performance table of the multiclass classification model of oral bioavailability, Table S5a: Performance table of the regression model of VD_ss_, Table S5b: Cross validation performance table of the regression model of VD_ss_, Table S6a. Performance table of the classification model of VD_ss_, Table S6b: Cross validation performance table of the classification model of VD_ss_, Table S7a: Performance table of the multiclass classification model of VD_ss_, Table S7b: Cross validation performance table of the multiclass classification model of VD_ss_, Table S8: Table of the best structural alerts found for the multiclass prediction of oral bioavailability, Table S9: Table of the best structural alerts found for the multiclass prediction of VD_ss_, Figure S1a: UMAP representation of the chemical space in a 2D map projection for the oral bioavailability dataset. Each point represents a chemical, and its color encodes the corresponding F% value, ranging from red for low oral bioavailability to blue for high oral bioavailability; 32% of chemicals have F% values below 30 (F%), 45% above 60 (F%), and 23% between 30 (F%) and 60 (F%), Figure S1b: UMAP representation of the chemical space in a 2D map projection for the VD_ss_ dataset. Points are colored considering the Log-transformed VD_ss_ values from red to blue for low to high VD_ss_; 37% of chemicals have VD_ss_ values below 0.6 L·kg^−1^, 16% above 5 L·kg^−1^, 47% between 0.6 L·kg^−1^, and 5 L·kg^−1^, Figure S2: General protocol applied for the development and evaluation of predictive models for the prediction of oral bioavailability and VD_ss_, Figure S3: Predicted vs. true oral bioavailability values on the 405 chemicals of the validation set. Red and blue dashed lines correspond to a 10% and 20% error, respectively, Figure S4: Predicted vs. true VD_ss_ values on the 405 chemicals of the validation set. Red and blue dashed lines correspond to a 2-fold and 3-fold error, respectively, Figure S5: Boxplot of the predictions for oral bioavailability, VD_ss_ and elimination half-life for a set of EDC categorized by chemical category, Figure S6: Plot of the predicted values against the hat values for the (a) oral bioavailability R-CatBoost model and the (b) VD_ss_ R-RF models, Table S10: Table of the best molecular descriptors for the best model of regression of oral bioavailability, Table S11: Table of the best molecular descriptors for the best model of regression of VD_ss_, Table S12: Table of models’ regression prediction on a set of toxicological and environmental chemical list of interest, Table S13: Table of models’ regression prediction on a list of endocrine disruptors, Table S14: Table of models’ regression prediction with molecular descriptors on a set of toxicological and environmental chemical list of interest, Table S15: Dataset for oral bioavailability, Table S16: Dataset for VD_ss_, File S1: QMRF VD_ss_, File S2: QMRF Oral bioavailability. References [63,64,80,81,82] are cited in Supplementary Materials.

## Abbreviations

The following abbreviations are used in this manuscript:

R	Regression
BC	Binary Classification
MC	Multiclass Classification
CV	Cross Validation
TK	Toxicokinetics
PK	Pharmacokinetics
GMFE	Geometric Mean Fold Error
QSAR	Quantitative Structure–Activity Relationship
VD	Volume of Distribution
VD_ss_	Volume of Distribution at Steady State
EDC	Endocrine-Disrupting Chemicals
SE	Sensitivity
SP	Specificity
BA	Balanced Accuracy
RMSE	Root Mean Squared Error
MAE	Mean Absolute Error
3-NN	Three Nearest Neighbors
AD	Applicability Domain
UMAP	Uniform Manifold Approximation and Projection for Dimension Reduction
