# Supplementary material for: QSAR Models for Predicting Oral Bioavailability and Volume of Distribution and Their Application in Mapping the TK Space of Endocrine Disruptors
Source: J Xenobiot. 2025 Oct 15;15(5):166. doi: 10.3390/jox15050166 (PMC12565085; doi:10.3390/jox15050166)
Supplement: Supplementary file 1 [file jox-15-00166-s001.zip › jox-3829321-File S2-QMRF Oral bioavailability.pdf]

Annex I –(Q)SAR model reporting format

(QMRF) v.2.1

QMRF v.2.1 is a minor update of the QMRF template, as it only concerns the description of the QMRF fields. The only exception is Section 10, which has been entirely removed. This section referred to the JRC QSAR Model Database, which is not updated anymore.

The update is based on the version 2.0[1].

	**Element**	**Explanation**
**1.**	**QSAR identifier**	
1.1.	QSAR identifier (title)	Oral bioavailability (INERIS/Université Paris Cité/Inserm) (version 1.0.1)
1.2	Other related models	[1–7]
1.3.	Software coding the model	python 3.9.7
**2.**	**General information**	

[1] Triebe, J., Worth, A., Janusch Roi, A. and Coe, A., JRC QSAR Model Database: EURL ECVAM DataBase service on ALternative Methods to animal experimentation: To promote the development and uptake of alternative and advanced methods in toxicology and biomedical sciences: User Support & Tutorial, EUR 28713 EN, Publications Office of the European Union, Luxembourg, 2017, ISBN 978-92-79-71406-1, doi:10.2760/905519, JRC107491.

Unclassified

2.0	Abstract	Toxicokinetics (TK) refers to the study of absorption, distribution metabolism, and excretion of xenobiotics in the organism [8]. Whose prediction is important for the characterization of TK propensities of potential drug candidates and for the assessment of chemical risk It is essential for safety assessment and for a comprehensive description of chemical toxicity. To provide decision-makers in the field of drug discovery and chemical risk assessment we developed machine learning QSAR models to predict oral bioavailability.
2.1.	Date of QMRF	11 February 2025
2.2.	QMRF author(s) and contact details	Guillaume Ollitrault, Inserm U1133, CNRS UMR 8251, Université de Paris Cité, Paris, France. guillaume.ollitrault@inserm.fr<br>Olivier Taboureau, Inserm U1133, CNRS UMR 8251, Université de Paris Cité, Paris, France. olivier.taboureau@univ-paris-diderot.fr<br>Enrico Mombelli, Institut National de l'Environnement Industriel et des Risques (INERIS), Verneuil en Halatte, France. Enrico.MOMBELLI@ineris.fr
2.3.	Date of QMRF update(s)	NA
2.4.	QMRF update(s)	NA
2.5.	Model developer(s) and contact details	[1] Guillaume Ollitrault, Inserm U1133, CNRS UMR 8251, Université de Paris Cité, Paris, France. guillaume.ollitrault@inserm.fr<br>[2] Olivier Taboureau, Inserm U1133, CNRS UMR 8251, Université de Paris Cité, Paris, France. olivier.taboureau@univ-paris-diderot.fr<br>[3] Enrico Mombelli, Institut National de l'Environnement Industriel et des Risques (INERIS), Verneuil en Halatte, France. Enrico.MOMBELLI@ineris.fr
2.6.	Date of model development and/or publication	11 February 2025
2.7.	Reference(s) to main scientific papers and/or software package	Ollitrault G., Marzo M., Roncaglioni A., Taboureau O., Mombelli E,. (2025) "QSAR models for Oral Bioavailability and volume of distribution and their application to the mapping of the TK space of Endocrine Disruptors"
2.8.	Availability of information about the model	The model is non-proprietary: full description of the model algorithm is available (python scripts).Training and test sets are available as supplementary material of original research article together with molecular descriptor values for each modelled chemical
2.9.	Availability of another QMRF for exactly the same model	NA
**3**	**Defining the endpoint - OECD Principle 1: "A DEFINED ENDPOINT"**	**PRINCIPLE 1: "A DEFINED ENDPOINT". ENDPOINT refers to any physicochemical, biological, or environmental property/activity/effect that can be measured and therefore modelled. The intent of PRINCIPLE 1 (a (Q)SAR should be associated with a defined endpoint) is to ensure clarity in the endpoint being predicted by a given model, since a given endpoint could be determined by different experimental protocols and under different experimental conditions. It is therefore important to identify the experimental system and test conditions that is being modelled by the Q)SAR.**
3.1.	Species	Homo Sapiens
3.2.	Endpoint	5. Toxicokinetics. Human Oral Absorption

3.3	Comment on endpoint	Oral bioavailability measures the fraction of an orally administered drug that reaches the systemic circulation (F%). It is calculated considering the relationship between plasma chemical concentration vs. time after both intravenous and oral administration. The oral bioavailability is computed as the percentage of the dose area under the curve of the concentration of chemicals in the plasma after oral administration divided by the dose area under the curve of the concentration of drugs in the plasma after intravenous administration [9].
3.4.	Endpoint units	Regression : % of drug that reaches the systemic circulation
3.5.	Dependent variable	NA
3.6.	Experimental protocol	Experimental protocols were described by Aungst, B. et al. (2017)[10]. Oral bioavailability were retrieved from multiple sources including OCHEM [11], CHEMBL [12] the article by Min Wei et.al [1], Falcón-Cano et al. [2], Varma et al. [13] and from the adme 2010 database [5]. In total, 1618 chemicals with associated oral bioavailability values were retrieved and curated and respectively 1712, 1649 chemicals with known classified F% values as 50% threshold or 30%-60% thresholds.
3.7.	Endpoint data quality and variability	According to Wang et al [14] the RMSE of experimental measurements error is 14.5%.
**4**	**Defining the algorithm - OECD Principle 2 : "AN UNAMBIGUOUS ALGORITHM"**	**PRINCIPLE 2: "AN UNAMBIGUOUS ALGORITHM". The (Q)SAR estimate of an endpoint is the result of applying an ALGORITHM to a set of structural parameters which describe the chemical structure. The intent of PRINCIPLE 2 (a (Q)SAR should be associated with an unambiguous algorithm) is to ensure transparency in the model algorithm that generates predictions of an endpoint from information on chemical structure and/or physicochemical properties. In this context, algorithm refers to any mathematical equation, decision rule or output approach.**
4.1.	Type of model	Statistical QSAR model using a CatBoost) algorithm as the quantitative classification.
4.2.	Explicit algorithm	CatBoost [15] is an optimized gradient boosting library built to design highly efficient parallel tree boosting models where each subsequent tree attempts to correct the errors made by the previous tree. It uses ordered boosting, and is designed to handle categorical data.<br>Model was optimized on the training set with grid search optimizing the "depth", "l2_leaf_reg" and the "learning_rate". The best model resulted in a "depth" of 6, a "l2_leaf_reg" of 5 and a "learning_rate" of 0.1.
4.3.	Descriptors in the model	66 molecular descriptors mordred (v1.1.1) [16] molecular descriptors were retained covering physicochemical properties of the molecules.<br>Molecular descriptors:<br>MID_O;ATSC1c;ATSC0c;GATS1se;CIC0;TopoPSA;AETA_eta;NsOH;ATS0are;EState_VSA8;GATS2are;SMR_VSA1;RPCG;BIC0;nHBDon;ATSC1d;BCUTd-1l;PEOE_VSA1;SlogP_VSA2;AATSC1s;RNCG;AATS2s;mZagreb1;GATS1are;AATS0s;AATS3i;VSA_EState2;BCUTare-1h;GATS1Z;MATS1are;BCUTs-1h;ATSC1are;Xpc-4dv;BCUTi-1h;ATSC0p;ATSC1s;ETA_dEpsilon_D;VSA_EState3;ATS0p;GATS2dv;VSA_EState1;EState_VSA1;SMR_VSA5;AATSC0s;MIC0;BCUTare-1l;ETA_dEpsilon_B;VSA_EState9;ATSC2i;AMID_X;EState_VSA10;MATS2are;GATS2i;ATSC1dv;AATSC2s;ATSC2p;SaaaC;IC3;Xc-6dv;MID_h;JGI9;BCUTse-1l;AMID_O;ATSC1p;ATSC1se;BCUTZ-1l

4.4.	Descriptor selection	The VSURF [17] algorithm was applied to select and retain the most informative molecular descriptors. The R package VSURF allows to identify most informative molecular descriptors using random forest importance scores based on permutation and using a stepwise forward strategy that selects the variables of the most accurate models. VSURF identifies two sets of molecular descriptors: the interpretation and the prediction level. We selected the interpretation set as it contains the most molecular descriptors.
4.5.	Algorithm and descriptor generation	We used Mordred molecular descriptors [16] as a function of normalized SMILES codes (see 6.6)
4.6.	Software name and version for descriptor generation	Mordred descriptors v1.1.1 [16]
4.7.	Chemicals/Descriptors ratio	Topliss ratio of 18:1
**5**	**Defining the applicability domain - OECD Principle 3: "A DEFINED DOMAIN OF APPLICABILITY"**	**PRINCIPLE 3: "A DEFINED DOMAIN OF APPLICABILITY". APPLICABILITY DOMAIN refers to the response and chemical structure space in which the model makes predictions with a given reliability. Ideally the applicability domain should express the structural, physicochemical and response space of the model. The CHEMICAL STRUCTURE (x variable) space can be expressed by information on physicochemical properties and/or structural fragments. The RESPONSE (y variable) can be any physicochemical, biological or environmental effect that is being predicted. According to PRINCIPLE 3 a (Q)SAR should be associated with a defined domain of applicability. Section 5 can be repeated (e.g., 5.a, 5.b, 5.c, etc) as many times as necessary if more than one method has been used to assess the applicability domain.**
5.1.	Description of the applicability domain of the model	Structural Alert based approach
5.2.	Method used to assess the applicability domain	The likelihood ratio (LR) for each structural alert (SA) developed with SARpy [18,19] associated with each predicted chemical was considered to gauge its precision in predicting a chemical in a certain category of the SARpy model. SARpy applicability domain model was trained on the training set categorised in 3 class low, medium and high values of oral bioavailability considering 30% and 60% thresholds. A chemical was deemed within the applicability domain if the LR of the structural alert responsible for the predicted compound exceeded 1.85. Threshold of 1.85 allowed to consider 65% of chemicals inside the AD of the external validation set.
5.3.	Software name and version for applicability domain assessment	Structural Alert based approach : SARpy
5.4.	Limits of applicability	Structural Alert based approach : A chemical was deemed within the applicability domain if the LR of the structural alert responsible for the predicted compound exceeded 1.85.

6	**Defining goodness-of-fit and robustness (internal validation) – OECD Principle 4: “APPROPRIATE MEASURES OF GOODNESS-OF-FIT, ROBUSTENESS AND PREDICTIVITY”**	**PRINCIPLE 4: “APPROPRIATE MEASURES OF GOODNESS-OF-FIT, ROBUSTENESS AND PREDICTIVITY”. PRINCIPLE 4 expresses the need to perform validation to establish the performance of the model. GOODNESS-OF-FIT and ROBUSTNESS refer to the internal model performance.**
6.1.	Availability of the training set	It is available as supporting information of the cited article (see 2.7)
6.2.	Available information for the training set	Available information :Chemical names (common names and/or IUPAC names); SMILES
6.3.	Data for each descriptor variable for the training set	It is available as supporting information of the cited article (see 2.7)
6.4.	Data for the dependent variable for the training set	It is available as supporting information of the cited article (see 2.7)
6.5.	Other information about the training set	Molecules were assigned to the training set by sorting the chemicals based on their oral bioavailability values. Every fourth chemical was excluded from the training set and included in the validation set
6.6.	Pre-processing of data before modelling	All the chemicals were mapped to their pubchem Compound ID (CID) in order to have standardized chemical structures according to the pubchem protocol [20] (i.e normalization of the representation, implicit hydrogens atom valence, tautomeric form representation, etc …). The pubchem CID was retrieved according to the available SMILES, CAS RN, name, InChI available from the source database. In the case of chemicals with ions the largest fragment was considered. This standardization allowed us to identify duplicate chemicals for which we computed the mean F% values. Duplicate chemicals with a difference in standard deviation of F% greater than 20, as well as F% values exceeding 100 or falling below 0, were excluded from the dataset. Only processed data is provided as supporting information of the cited article (see2.7).
6.7.	Statistics for goodness-of-fit	- Regression :

RMSE	2.08
R^2	0.99
Q2F3	0.39

- Classification (50% Threshold) :
  The regression model was evaluated for it’s ability to predict dichotomized class of low or high oral bioavailability values considering a 50% thresholds.

Sensitivity	1.00
Specificity	0.97
Balanced accuracy	0.99

- Multiclass classification (30%-60% Threshold) :
  The regression model was evaluated for it’s ability to predict multi class of low, medium or high oral bioavailability values considering a 30% and 60% thresholds.

Sensitivity (<30%)	0.99
Specificity (<30%)	1
Balanced accuracy (<30%)	0.99
Sensitivity [30%-60%]	0.97
Specificity [30%-60%]	0.97
Balanced accuracy [30%-60%]	0.97

<table>
<tr><td></td><td></td><td>
<table>
<tr><td>Sensitivity (>60%)</td><td>0.97</td></tr>
<tr><td>Specificity (>60%)</td><td>1</td></tr>
<tr><td>Balanced accuracy (>60%)</td><td>0.98</td></tr>
<tr><td>Macro Sensitivity</td><td>0.98</td></tr>
<tr><td>Macro Specificity</td><td>0.99</td></tr>
<tr><td>Macro Balanced accuracy</td><td>0.98</td></tr>
<tr><td>Micro Sensitivity</td><td>0.97</td></tr>
<tr><td>Micro Specificity</td><td>0.99</td></tr>
<tr><td>Micro Balanced accuracy</td><td>0.98</td></tr>
</table>
</td></tr>
<tr><td>6.8.</td><td>Robustness - Statistics obtained by leave-one-out cross-validation</td><td>NA</td></tr>
<tr><td>6.9.</td><td>Robustness - Statistics obtained by leave-many-out cross-validation</td><td>NA</td></tr>
<tr><td>6.10.</td><td>Robustness - Statistics obtained by Y-scrambling</td><td>NA</td></tr>
<tr><td>6.11.</td><td>Robustness - Statistics obtained by bootstrap</td><td>5-fold cross validation :
- Regression :
<table>
<tr><td>RMSE</td><td>26.71 ± 0.98</td></tr>
<tr><td>R²</td><td>0.38 ± 0.04</td></tr>
<tr><td>MAE</td><td>20.90 ± 0.81</td></tr>
<tr><td>Q²F3</td><td>0.34 ± 0.05</td></tr>
</table>
- Classification (50%Threshold) :
The regression model was evaluated for its ability to predict dichotomized classes of low or high oral bioavailability values considering a 50% threshold.
<table>
<tr><td>Sensitivity</td><td>0.75 ± 0.03</td></tr>
<tr><td>Specificity</td><td>0.72 ± 0.03</td></tr>
<tr><td>Balanced accuracy</td><td>0.74 ± 0.02</td></tr>
</table>
- Multiclass classification (30%-60% Threshold) :
The regression model was evaluated for it's ability to predict multi class of low, medium or high oral bioavailability values considering a 30% and 60% threshold.
<table>
<tr><td>Sensitivity (<30%)</td><td>0.44 ± 0.05</td></tr>
<tr><td>Specificity (<30%)</td><td>0.93 ± 0.02</td></tr>
<tr><td>Balanced accuracy (<30%)</td><td>0.69 ± 0.02</td></tr>
<tr><td>Sensitivity [30%-60%]</td><td>0.63 ± 0.06</td></tr>
<tr><td>Specificity [30%-60%]</td><td>0.63 ± 0.03</td></tr>
<tr><td>Balanced accuracy [30%-60%]</td><td>0.74 ± 0.02</td></tr>
<tr><td>Sensitivity (>60%)</td><td>0.63 ± 0.04</td></tr>
<tr><td>Specificity (>60%)</td><td>0.84 ± 0.03</td></tr>
<tr><td>Balanced accuracy (>60%)</td><td>0.74 ± 0.02</td></tr>
</table>
</td></tr>
<tr><td>6.12.</td><td>Robustness - Statistics obtained by other methods</td><td>NA</td></tr>
</table>

Unclassified

7	**Defining predictivity (external validation) – OECD Principle 4: "APPROPRIATE MEASURES OF GOODNESS-OF-FIT, ROBUSTENESS AND PREDICTIVITY"**	**PRINCIPLE 4: "APPROPRIATE MEASURES OF GOODNESS-OF-FIT, ROBUSTENESS AND PREDICTIVITY". PRINCIPLE 4 expresses the need to perform validation to establish the performance of the model. PREDICTIVITY refers to the external model validation. Section 7 can be repeated (e.g., 7.a, 7.b, 7.c, etc) as many times as necessary if more validation studies need to be reported in the QMRF.**
7.1.	Availability of the external validation set	It is available as supporting information of the cited article (see 2.7)
7.2.	Available information for the external validation set	Available information : a) Chemical names (common names and/or IUPAC names); b) SMILES
7.3.	Data for each descriptor variable for the external validation set	NA
7.4.	Data for the dependent variable for the external validation set	It is available as supporting information of the cited article (see 2.7)
7.5.	Other information about the external validation set	External validation set with 405 compounds
7.6.	Experimental design of test set	Molecules in the external set were selected by sorting oral bioavailability values and we included every fourth chemical to this set, ensuring representative inclusion across the range of oral bioavailability values
7.7.	Predictivity - Statistics obtained by external validation	- Regression : (see tables below)

- Regression :

RMSE	25.86
R^2	0.42
Q2F3	0.39

- Classification (50% Threshold) :
  The regression model was evaluated for its ability to predict dichotomized classes of low or high oral bioavailability values considering a 50% threshold.

Sensitivity	0.78
Specificity	0.76
Balanced accuracy	0.77

- Multiclass classification (30%-60% Threshold) :
  The regression model was evaluated for it's ability to predict multi class of low, medium or high oral bioavailability values considering 30% and 60% threshold.

Sensitivity (<30%)	0.45
Specificity (<30%)	0.91
Balanced accuracy (<30%)	0.68
Sensitivity [30%-60%]	0.58
Specificity [30%-60%]	0.63
Balanced accuracy [30%-60%]	0.60
Sensitivity (>60%)	0.63
Specificity (>60%)	0.84
Balanced accuracy (>60%)	0.74
Macro Sensitivity	0.56
Macro Specificity	0.79

<table>
<tr><td></td><td></td><td><table><tr><td>Macro Balanced accuracy</td><td>0.68</td></tr><tr><td>Micro Sensitivity</td><td>0.56</td></tr><tr><td>Micro Specificity</td><td>0.78</td></tr><tr><td>Micro Balanced accuracy</td><td>0.67</td></tr></table></td></tr>
<tr><td>7.8.</td><td>Predictivity - Assessment of the external validation set</td><td>NA</td></tr>
<tr><td>7.9.</td><td>Comments on the external validation of the model</td><td>The external set was composed of molecules not used during the training and the hyperparametrisation of the model.</td></tr>
<tr><td>8</td><td>Providing a mechanistic interpretation - OECD Principle 5: “A MECHANISTIC INTERPRETATION, IF POSSIBLE”</td><td>PRINCIPLE 5: “A MECHANISTIC INTERPRETATION, IF POSSIBLE”. According to PRINCIPLE 5, a (Q)SAR should be associated with a mechanistic interpretation, if possible.</td></tr>
<tr><td>8.1.</td><td>Mechanistic basis of the model</td><td>Complex molecular descriptors that retain topological and electrostatic information seem to be able to predict oral bioavailability.(See cited article (see 2.7)

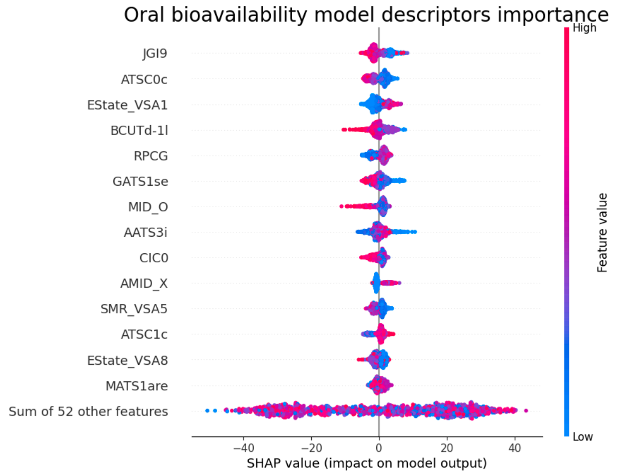

Figure. Summary plot obtained by using the SHAP package [21]. The plot shows the importance of the 15 most important molecular descriptors and their effect on the predictions for the CatBoost regression model. The plot enables the observation of the relationship between a molecular descriptor value and its impact on the prediction.

JGI9 (9-ordered mean topological charge), ATSC0c (centered moreau-broto autocorrelation of lag 0 weighted by gasteiger charge), Estate_VSA1 (Labute’s Approximate Surface Area EState indices and surface area), BCUTd-1l (first lowest eigenvalue of Burden matrix weighted by sigma electrons), MID_O (molecular ID on O atoms) molecular descriptors being of most importance in the model. For example high values of JGI9 tend to decrease oral bioavailability.</td></tr>
<tr><td>8.2.</td><td>A priori or a posteriori mechanistic interpretation</td><td>The Mechanical basis of the model was determined a posteriori (i.e. after modelling, by interpretation of the final model).</td></tr>
<tr><td>8.3.</td><td>Other information about the mechanistic interpretation</td><td>These molecular descriptors are consistent with those identified in previous models developed for oral bioavailability. For instance, the model by Wei et al. [1] highlighted SsOH (an E-state molecular descriptor), ATS5i (a topological structure molecular descriptor), and TopoPSA(NO) as the most important. Similarly, the model by Ma et al.</td></tr>
</table>

		[22] identified additional topological structure molecular descriptors, including TopoPSA and TopoPSA(NO), along with an E-state molecular descriptor (EState_VSA8) and the MID_O molecular descriptor, which is related to the identification and characterization of oxygen atoms in a molecule.
**9**	**Miscellaneous information**	
9.1.	Comments	NA
9.2.	Bibliography	1. Wei, M.; Zhang, X.; Pan, X.; Wang, B.; Ji, C.; Qi, Y.; Zhang, J.Z.H. HobPre: Accurate Prediction of Human Oral Bioavailability for Small Molecules. *J. Cheminformatics* **2022**, *14*, 1, doi:10.1186/s13321-021-00580-6.<br>2. Falcón-Cano, G.; Molina, C.; Cabrera-Pérez, M.Á. ADME Prediction with KNIME: Development and Validation of a Publicly Available Workflow for the Prediction of Human Oral Bioavailability. *J. Chem. Inf. Model.* **2020**, *60*, 2660–2667, doi:10.1021/acs.jcim.0c00019.<br>3. Venkatraman, V. FP-ADMET: A Compendium of Fingerprint-Based ADMET Prediction Models. *J. Cheminformatics* **2021**, *13*, 75, doi:10.1186/s13321-021-00557-5.<br>4. Xiong, G.; Wu, Z.; Yi, J.; Fu, L.; Yang, Z.; Hsieh, C.; Yin, M.; Zeng, X.; Wu, C.; Lu, A.; et al. ADMETlab 2.0: An Integrated Online Platform for Accurate and Comprehensive Predictions of ADMET Properties. *Nucleic Acids Res.* **2021**, *49*, W5–W14, doi:10.1093/nar/gkab255.<br>5. Tian, S.; Li, Y.; Wang, J.; Zhang, J.; Hou, T. ADME Evaluation in Drug Discovery. 9. Prediction of Oral Bioavailability in Humans Based on Molecular Properties and Structural Fingerprints. *Mol. Pharm.* **2011**, *8*, 841–851, doi:10.1021/mp100444g.<br>6. Kim, M.T.; Sedykh, A.; Chakravarti, S.K.; Saiakhov, R.D.; Zhu, H. Critical Evaluation of Human Oral Bioavailability for Pharmaceutical Drugs by Using Various Cheminformatics Approaches. *Pharm. Res.* **2014**, *31*, 1002–1014, doi:10.1007/s11095-013-1222-1.<br>7. Musther, H.; Olivares-Morales, A.; Hatley, O.J.D.; Liu, B.; Rostami Hodjegan, A. Animal versus Human Oral Drug Bioavailability: Do They Correlate? *Eur. J. Pharm. Sci.* **2014**, *57*, 280–291, doi:10.1016/j.ejps.2013.08.018.<br>8. Shanmugam, P.S.T.; Sampath, T.; Jagadeeswaran, I.; Bhalerao, V.P.; Thamizharasan, S.; V., K.; Saha, J.

		Toxicokinetics. In *Biocompatibility Protocols for Medical Devices and Materials*; Elsevier, 2023; pp. 175–186 ISBN 978-0-323-91952-4.<br>9. Drug Bioavailability. **2023**.<br>10. Aungst, B.J. Optimizing Oral Bioavailability in Drug Discovery: An Overview of Design and Testing Strategies and Formulation Options. *J. Pharm. Sci.* **2017**, *106*, 921–929, doi:10.1016/j.xphs.2016.12.002.<br>11. Sushko, I.; Novotarskyi, S.; Körner, R.; Pandey, A.K.; Rupp, M.; Teetz, W.; Brandmaier, S.; Abdelaziz, A.; Prokopenko, V.V.; Tanchuk, V.Y.; et al. Online Chemical Modeling Environment (OCHEM): Web Platform for Data Storage, Model Development and Publishing of Chemical Information. *J. Comput. Aided Mol. Des.* **2011**, *25*, 533–554, doi:10.1007/s10822-011-9440-2.<br>12. Gaulton, A.; Bellis, L.J.; Bento, A.P.; Chambers, J.; Davies, M.; Hersey, A.; Light, Y.; McGlinchey, S.; Michalovich, D.; Al-Lazikani, B.; et al. ChEMBL: A Large-Scale Bioactivity Database for Drug Discovery. *Nucleic Acids Res.* **2012**, *40*, D1100–D1107, doi:10.1093/nar/gkr777.<br>13. Varma, M.V.S.; Obach, R.S.; Rotter, C.; Miller, H.R.; Chang, G.; Steyn, S.J.; El-Kattan, A.; Troutman, M.D. Physicochemical Space for Optimum Oral Bioavailability: Contribution of Human Intestinal Absorption and First-Pass Elimination. *J. Med. Chem.* **2010**, *53*, 1098–1108, doi:10.1021/jm901371v.<br>14. Wang, J.; Krudy, G.; Xie, X.-Q.; Wu, C.; Holland, G. Genetic Algorithm-Optimized QSPR Models for Bioavailability, Protein Binding, and Urinary Excretion. *J. Chem. Inf. Model.* **2006**, *46*, 2674–2683, doi:10.1021/ci060087t.<br>15. Prokhorenkova, L.; Gusev, G.; Vorobev, A.; Dorogush, A.V.; Gulin, A. CatBoost: Unbiased Boosting with Categorical Features 2019.<br>16. Moriwaki, H.; Tian, Y.-S.; Kawashita, N.; Takagi, T. Mordred: A Molecular Descriptor Calculator. *J. Cheminformatics* **2018**, *10*, 4, doi:10.1186/s13321-018-0258-y.<br>17. Genuer, R.; Poggi, J.-M.; Tuleau-Malot, C. VSURF: An R Package for Variable Selection Using Random Forests. *R J.* **2015**, *7*, 19, doi:10.32614/RJ-2015-018.<br>18. Ferrari, T.; Gini, G.; Golbamaki Bakhtyari, N.; Benfenati, E. Mining Toxicity

		Structural Alerts from SMILES: A New Way to Derive Structure Activity Relationships. In Proceedings of the 2011 IEEE Symposium on Computational Intelligence and Data Mining (CIDM); IEEE: Paris, France, April 2011; pp. 120–127.<br>19. Ferrari, T.; Cattaneo, D.; Gini, G.; Golbamaki Bakhtyari, N.; Manganaro, A.; Benfenati, E. Automatic Knowledge Extraction from Chemical Structures: The Case of Mutagenicity Prediction. *SAR QSAR Environ. Res.* **2013**, *24*, 365–383, doi:10.1080/1062936X.2013.773376.<br>20. Hähnke, V.D.; Kim, S.; Bolton, E.E. PubChem Chemical Structure Standardization. *J. Cheminformatics* **2018**, *10*, 36, doi:10.1186/s13321-018-0293-8.<br>21. Lundberg, S.M.; Lee, S.-I. A Unified Approach to Interpreting Model Predictions. In *Advances in Neural Information Processing Systems 30*; Guyon, I., Luxburg, U.V., Bengio, S., Wallach, H., Fergus, R., Vishwanathan, S., Garnett, R., Eds.; Curran Associates, Inc., 2017; pp. 4765–4774.<br>22. Ma, L.; Yan, Y.; Dai, S.; Shao, D.; Yi, S.; Wang, J.; Li, J.; Yan, J. Research on Prediction of Human Oral Bioavailability of Drugs Based on Improved Deep Forest. *J. Mol. Graph. Model.* **2024**, *133*, 108851, doi:10.1016/j.jmgm.2024.108851.
9.3	Supporting information	NA
