# Supplementary material for: QSAR Models for Predicting Oral Bioavailability and Volume of Distribution and Their Application in Mapping the TK Space of Endocrine Disruptors
Source: J Xenobiot. 2025 Oct 15;15(5):166. doi: 10.3390/jox15050166 (PMC12565085; doi:10.3390/jox15050166)
Supplement: Supplementary file 1 [file jox-15-00166-s001.zip › jox-3829321-Table_S8-S11_Figure_S1-S6.pdf]

Journal of
Xenobiotics

MDPI

Supplementary Materials: QSAR models for Predicting Oral Bioavailability and Volume of Distribution and Their Application in Mapping the TK Space of Endocrine Disruptors

Guillaume Ollitrault, Marco Marzo, Alessandra Roncaglioni, Olivier Taboureau and Enrico Mombelli

Supplementary Data : Chemical space

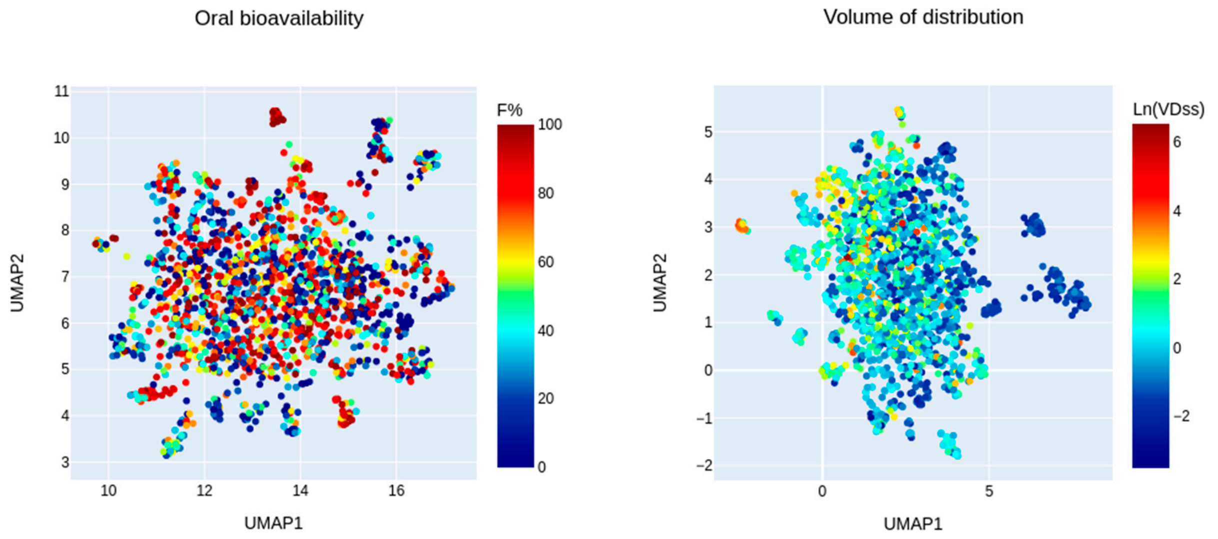

(a) **(b)**

Figure S1. (a) UMAP representation of the chemical space in a 2D map projection for the oral bioavailability dataset. Each point represents a chemical, and its color encodes the corresponding F% value, ranging from red for low oral bioavailability to blue for high oral bioavailability. 32% of chemicals have an F% values below 30 (F%), 45% above 60 (F%) and 23% between 30 (F%) and 60 (F%). **(b)** UMAP representation of the chemical space in a 2D map projection for the VD_{ss} dataset. Points are colored considering the Log transformed VD_{ss} values ($lnVD_{ss}$) from red to blue for low to high VD_{ss}. 37% of chemicals have a VD_{ss} values below 0.6 $L.Kg^{-1}$, 16% above 5 $L.Kg^{-1}$, 47% between 0.6 $L.Kg^{-1}$, and 5 $L.Kg^{-1.}$

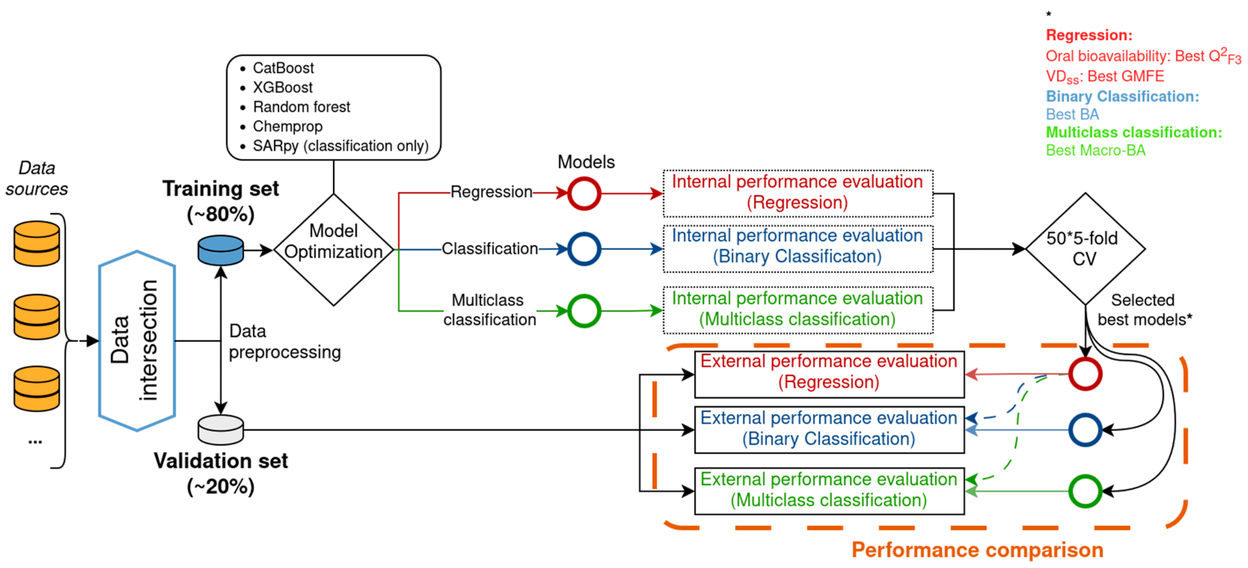

Figure S2: General protocol applied for the development and evaluation of predictive models for the prediction of oral bioavailability and VD_{ss}. The asterisk (*) indicates which metrics were used to select the best model.

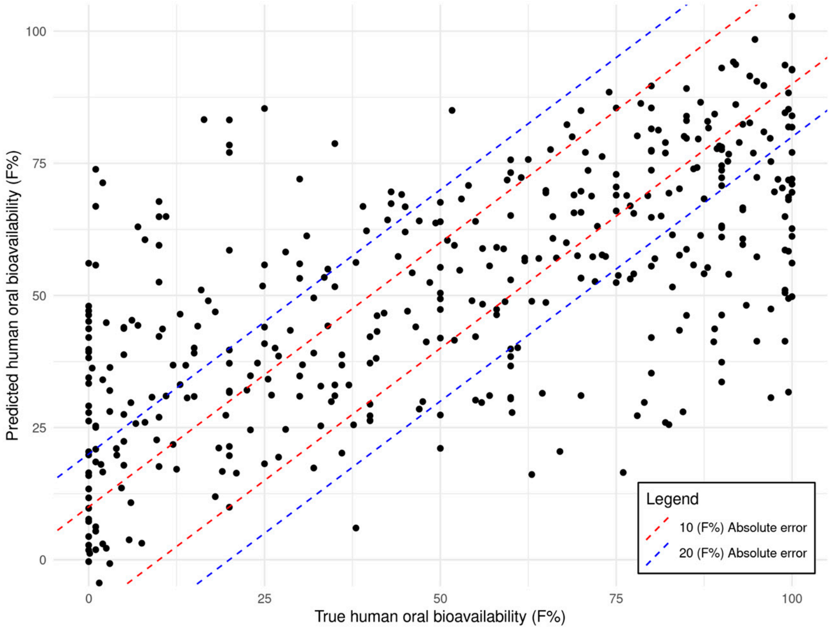

Figure S3: Predicted vs true oral bioavailability values on the 405 chemicals of the validation set. Red and blue dashed lines correspond to a 10 and 20 F% absolute error respectively.

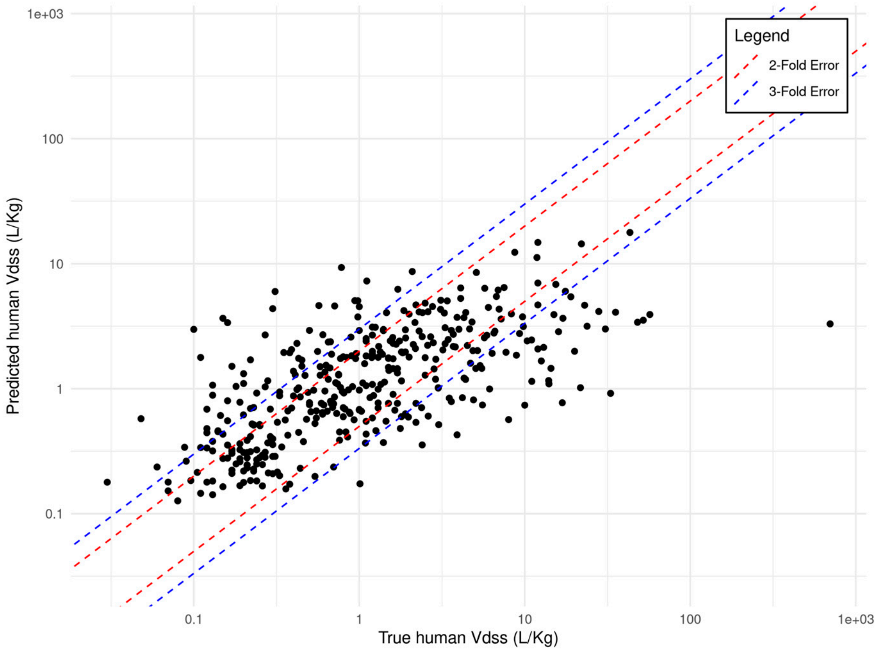

Figure S4: Predicted vs true Vd_{ss} values on the 405 chemicals of the validation set. Red and blue dashed lines correspond to a 2-fold and 3-fold absolute error respectively.

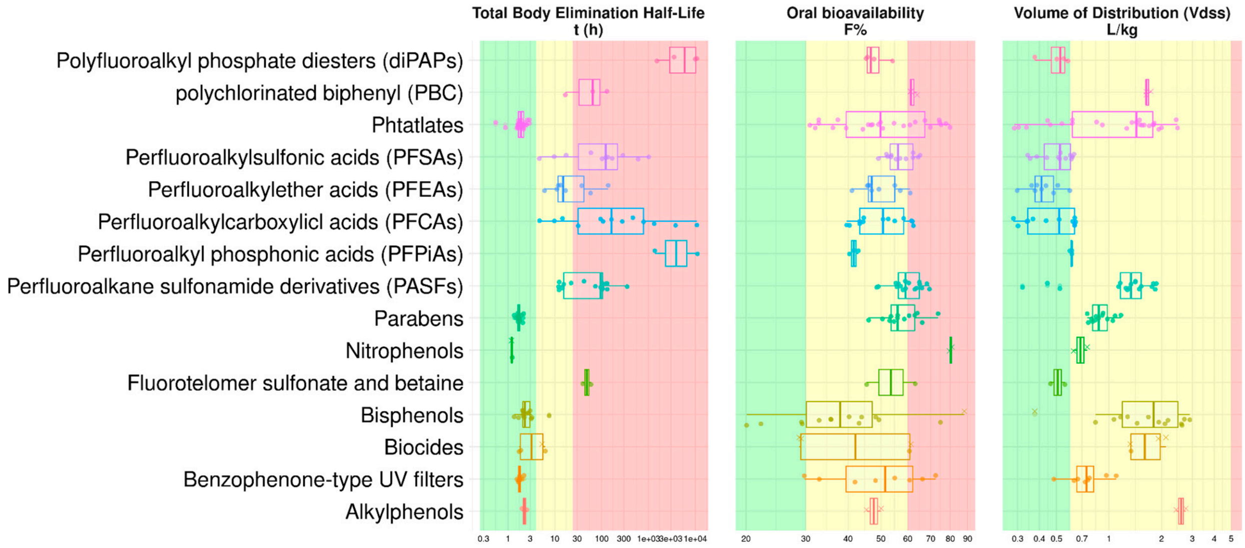

Figure S5: Boxplot of the predictions for oral bioavailability, VD_{ss} and elimination half-life for a set of EDC categorized by chemical category.

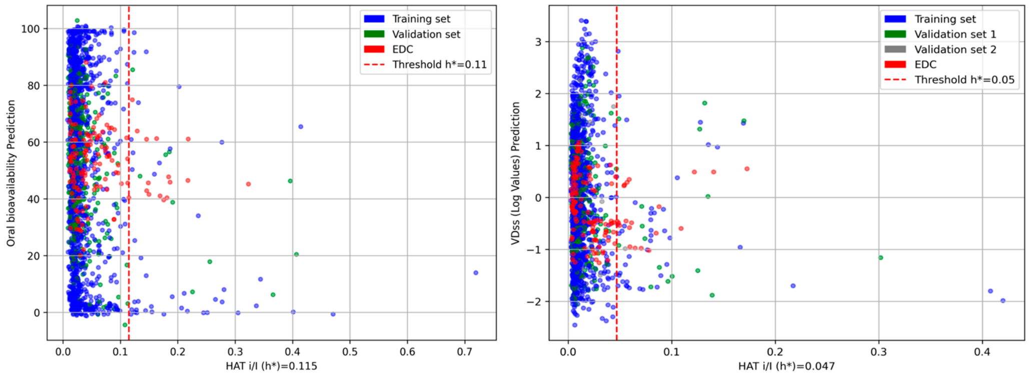

(a) **(b)**

Figure S6: Insubria plot for the (a) oral bioavailability R-CatBoost model and the (b) VD_{ss} R-RF models. Basically, the plot of *hat* values vs. predicted values for the different datasets are presented. For each chemical, the hat value (h_{ii}) is defined by the leverage approach from hat matrix (for the structural domain), and by the identification of response outliers (compounds with cross- validated standardized residuals greater than 2.5 standard deviation units). The threshold h* are calculated based on *compounds with* h_{ii} *greater than* $3p'/n$ *(h*)*, where p' is the number of the model variables plus one, and n is the number of training compounds. The chemicals with a $h_{ii} > h^*$ are considered outside the applicability domain. More information about the Insubria plot can be fine at DOI : 10.1002/minf.201200075.

Supplementary Materials: Machine learning algorithms

- Random Forest is an ensemble learning method that combines the output of multiple decision trees to make a prediction. It can be useful for the development of classification as well as regression models.
- XGBoost and CatBoost are two optimized gradient boosting libraries designed for highly efficient parallel tree boosting, where each successive tree corrects the errors of the previous one.
- CatBoost uses ordered boosting that allows to train a model by performing a permutation on a subset of data while calculating residuals on another subset. The CatBoost library can handle categorical data.
- Chemprop is a python package performing directed message passing neural networks (D-MPNN) designed to treat molecular properties. D-MPNN is a class of graph-convolutional neural networks where chemicals are represented as edges and vertices. The model works in two steps: the message passing phase which transforms the chemical into a neural representation and the readout phase which makes the prediction considering the neural representation of the chemical. The chemprop package allows to compute morgan fingerprints or RDKit 2D fingerprints as additional molecular descriptors to improve the performance of the generated models. We choose to include the RDKit 2d molecular descriptors in our modeling process with an ensemble size of 5 (number of developed models whose predictions are averaged).
- SARpy is a modelling approach implemented in python to facilitate the modeling of structure-activity relationship models. It recursively mines every substructure in a training set. Each substructure is explored as a potential structural alert on the training set by assessing its predictive power. When a specific structural alert is found within a query chemical, the activity associated with the alert is then attributed to this chemical to predict the biological property of interest. The SARpy method is designed only for classification.

Table S8: Table of the best structural alerts found for the multiclass prediction of oral bioavailability.

SMARTS	Class prediction	Training LR	Image
NCC(O)OC	<30%	inf	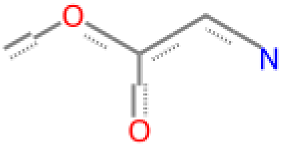
C[N+](C)(C)CCCC	<30%	inf	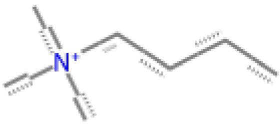
C(CC2CCCCC2)C(O)C(O)	<30%	inf	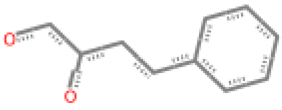
O=C(O)CCNC(=O)	<30%	inf	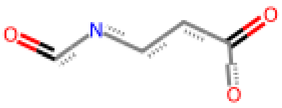

P(O)(O)CP(O)O	<30%	inf	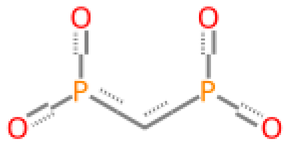
CCC1CCCC(O)O1	<30%	inf	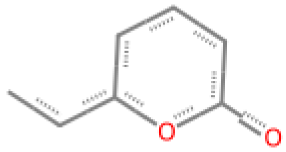
Cn1cc(C(=O))cc2ccccc12	>60%	inf	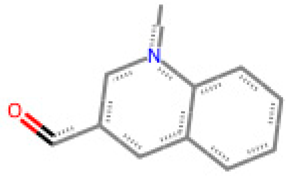
c1ccc(S(=O)(=O)NC(=O))cc1	>60%	inf	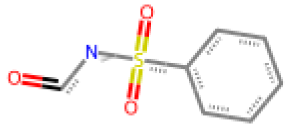
C(C(=O)N)(C(=O)N)	>60%	inf	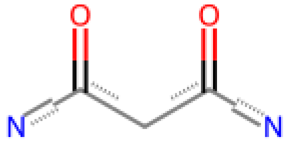

C2CCc3c(O)cccc3O2	<30%	inf	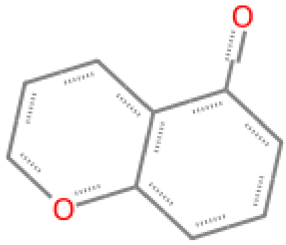
CCCCCNC(CCN)C	<30%	inf	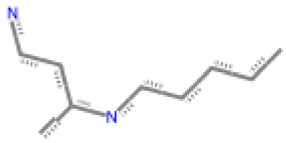
c1ccc[n+](C)c1	<30%	inf	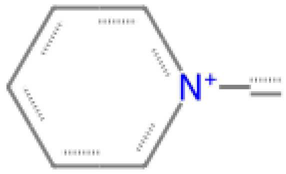
C(=O)c1cnc3cccc c13	>60%	inf	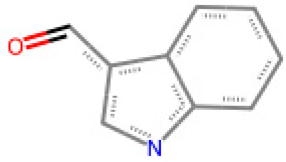
COc1ccc2c(c1)cc n2	>60%	inf	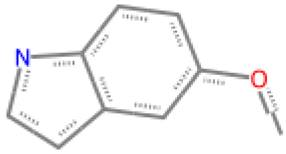

Table S9: Table of the best structural alerts found for the multiclass prediction of VD_{ss}.

SMARTS	Class	Training LR	Image
C2CCN2CC	<0.6	inf	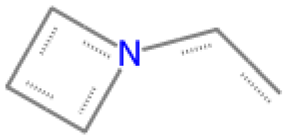
C(=O)C(C)NC(=O)C(N)CCC(=O)	<0.6	inf	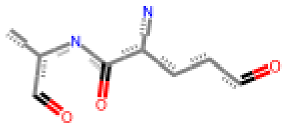
O=CCN(CCN(CC)CC)CC	<0.6	inf	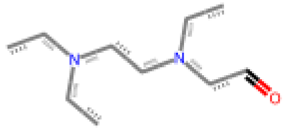
C(=O)c1c(I)c(NC(=O)C)c(I)cc1I	<0.6	inf	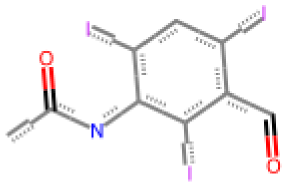
c1ccc(S(=O)O)cc1	<0.6	inf	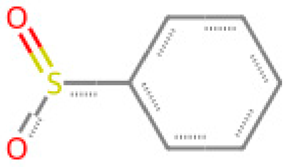

OC2OC(CN)CCC2	<0.6	inf	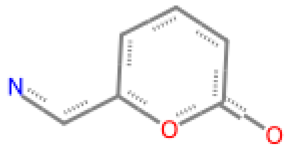
SCCCC(=O)	<0.6	inf	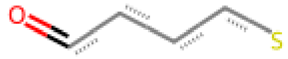
CCOC(=O)C(C)CC(C)	>5	inf	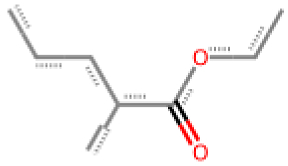
C(C(=O))c1ccc(C)cc1	<0.6	inf	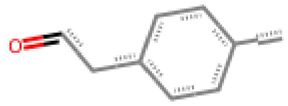
CC(C)NCC(O)COc1ccccc1	>5	inf	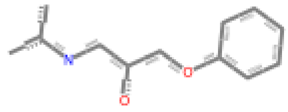

CCN(CC)CCNC(=O)c1ccccc1OC	>5	inf	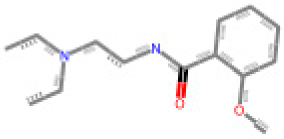
O=C(O)c1cn(C2CC2)c2ccccc2c1=O	>5	inf	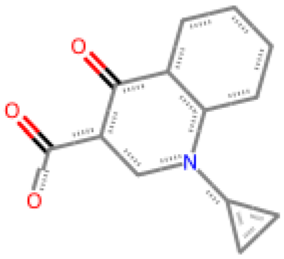
CCC(c2cccc(OC)c2)C(C)C	>5	inf	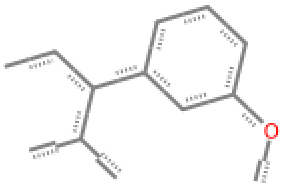
CC(Cc1ccccc1F)	>5	inf	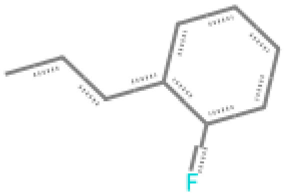
C(NC(=O))C(C)(C)C	>5	inf	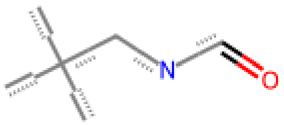

C1=NCCN1	>5	inf	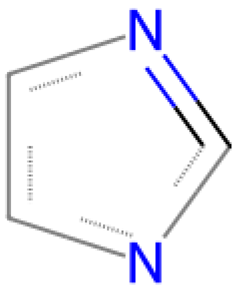
CS(=O)c2nc3ccccc3[nH]2	<0.6	inf	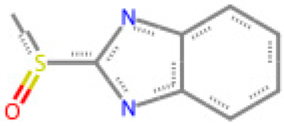

Table S10: Table of the best molecular descriptors for the best model of regression of oral bioavailability.

Molecular Descriptor name	Description	Example molecule with low value	Example molecule with high value
jGI9	9-ordered mean topological charge	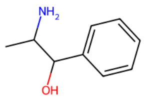	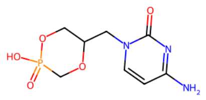
ATSC0c	centered moreau-broto autocorrelation of lag 0 weighted by gasteiger charge	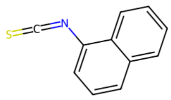	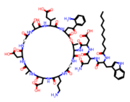
EState_VSA1	EState VSA Descriptor 1 (-inf < x < -0.39)	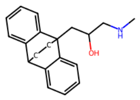	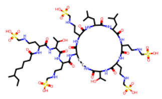
BCUTd-1l	first lowest eigenvalue of Burden matrix weighted by sigma electrons	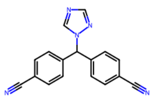	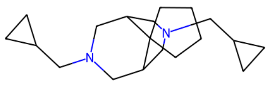
RPCG	relative positive charge	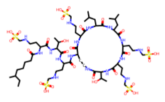	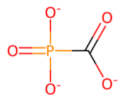

GATS1se	geary coefficient of lag 1 weighted by sanderson EN	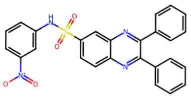	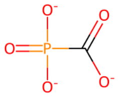
MID_O	molecular ID on O atoms	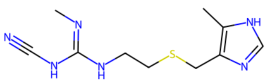	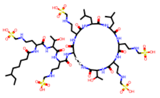
AATS3i	averaged moreau-broto autocorrelation of lag 3 weighted by ionization potential	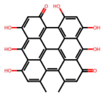	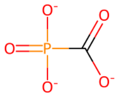
CIC0	0-ordered complementary information content	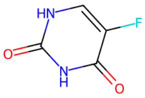	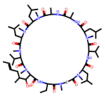
AMID_X	averaged molecular ID on halogen atoms	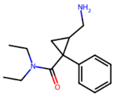	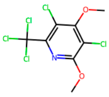
SMR_VSA5	MOE MR VSA Descriptor 5 (2.45 <= x < 2.75)	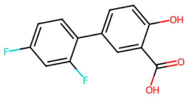	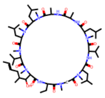
ATSC1c	centered moreau-broto autocorrelation of lag 1 weighted by gasteiger charge	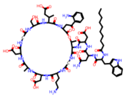	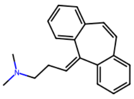
EState_VSA8	EState VSA Descriptor 8 (2.05 <= x < 4.69)	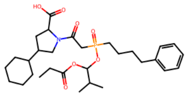	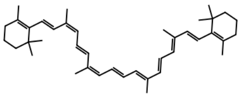
MATS1are	moran coefficient of lag 1 weighted by allred-rocow EN	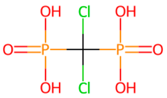	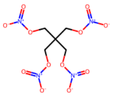

Table S11: Table of the best molecular descriptors for the best model of regression of VD_{ss}.

Molecular Descriptor name	Description	Example molecule with low value	Example molecule with high value
nAcid	acidic group count	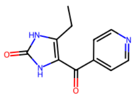	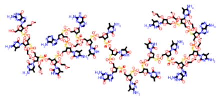
nBase	basic group count	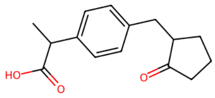	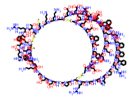
SLogP	Wildman-Crippen LogP	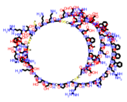	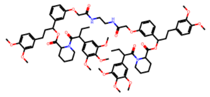
AATS1p	averaged moreau-broto autocorrelation of lag 1 weighted by polarizability	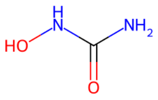	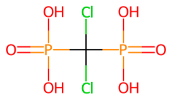
BCUTp-1l	first lowest eigenvalue of Burden matrix weighted by polarizability	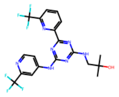	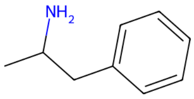
BCUTd-1l	first lowest eigenvalue of Burden matrix weighted by sigma electrons	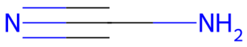	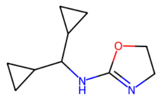
AMID_C	averaged molecular ID on C atoms	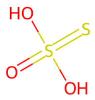	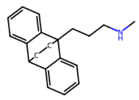
AATSC1c	averaged and centered moreau-broto autocorrelation of lag 1 weighted by gasteiger charge	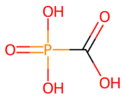	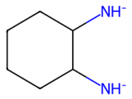
FilterItLogS	Filter-it™ LogS	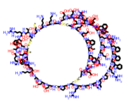	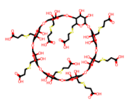

PEOE_VSA7	MOE Charge VSA Descriptor 7 (-0.05 <= x < 0.00)	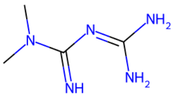	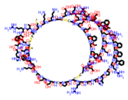
AMID_O	averaged molecular ID on O atoms	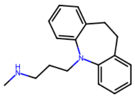	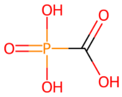
NaasC	number of aasC	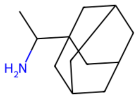	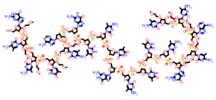
VSA_EState3	VSA EState Descriptor 3 (5.00 <= x < 5.41)	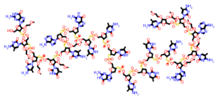	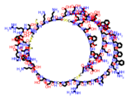
AATSC2s	averaged and centered moreau-broto autocorrelation of lag 2 weighted by intrinsic state	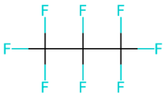	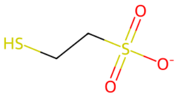
